# Deregulation of sertoli and leydig cells function in patients with klinefelter syndrome as evidenced by testis transcriptome analysis

**DOI:** 10.1186/s12864-015-1356-0

**Published:** 2015-03-07

**Authors:** Marco D’Aurora, Alberto Ferlin, Marta Di Nicola, Andrea Garolla, Luca De Toni, Sara Franchi, Giandomenico Palka, Carlo Foresta, Liborio Stuppia, Valentina Gatta

**Affiliations:** Department of Psychological, Humanities and Territorial Sciences, School of Medicine and Health Sciences, “G.d’Annunzio” University, Via Dei Vestini 31, 66100 Chieti-Pescara, Italy; Functional Genetics Unit, Center of Excellence on Aging (Ce.S.I.), Via Dei Vestini 31, 66100 Chieti, Italy; Department of Medicine, Section of Endocrinology and Centre for Human Reproduction Pathology, University of Padova, Via Giustiniani 2, 35128 Padova, Italy; Department of Sperimental and Clinical Sciences, School of Medicine and Health Sciences, “G.d’Annunzio” University, Via dei Vestini 31, 66100 Chieti-Pescara, Italy; Department of Oral Health and Biotechnological Sciences, School of Medicine and Health Sciences, “G.d’Annunzio” University, Via dei Vestini 31, 66100 Chieti-Pescara, Italy

**Keywords:** Klinefelter Syndrome, Sertoli cells, Leydig cells, Testis transcriptome, Microarray, Male infertility

## Abstract

**Background:**

Klinefelter Syndrome (KS) is the most common abnormality of sex chromosomes (47,XXY) and represents the first genetic cause of male infertility. Mechanisms leading to KS testis degeneration are still not completely defined but considered to be mainly the result of germ cells loss. In order to unravel the molecular basis of global testis dysfunction in KS patients, we performed a transcriptome analysis on testis biopsies obtained from 6 azoospermic non-mosaic KS patients and 3 control subjects.

**Results:**

The analysis found that, compared to controls, KS patients showed the differential up- and down-expression of 656 and 247 transcripts. The large majority of the deregulated transcripts were expressed by Sertoli cells (SCs) and Leydig cells (LCs). Functional analysis of the deregulated transcripts indicated changes of genes involved in cell death, inflammatory response, lipid metabolism, steroidogenesis, blood-testis-barrier formation and maintenance, as well as spermatogenesis failure.

**Conclusions:**

Taken together, present data highlight the modulation of hundreds of genes in the somatic components of KS patient testis. The increased LCs steroidogenic function together with the impairment of inflammatory pathways and BTB structure, result in increased apoptosis. These findings may represent a critical roadmap for therapeutic intervention and prevention of KS-related testis failure.

**Electronic supplementary material:**

The online version of this article (doi:10.1186/s12864-015-1356-0) contains supplementary material, which is available to authorized users.

## Background

Klinefelter Syndrome (KS) is the most common abnormality of sex chromosomes, with a prevalence of 1:600-1:1000 in newborn males, even if this condition is generally diagnosed in the adulthood [1]. KS is characterized by the presence of a 47, XXY karyotype in about 80- 90% of patients, being the remaining cases represented by chromosome mosaicisms (e.g. 47, XXY/46, XY), additional sex chromosomes (e.g. 48, XXXY; 48, XXYY; 49, XXXXY) or X chromosome structural abnormalities (e.g. 47,X,iXq,Y) [2]. KS subjects show a variable wide phenotypic spectrum in the adulthood that however always includes primary testicular failure with reduced testicular volume, hypergonadotropic hypogonadism, and azoospermia or severe oligozoospermia in 90% and 10% of non-mosaic patients, respectively [3-5]. KS represents the main genetic cause of male infertility, being present in 15% of azoospermic men and 3% of all infertile men [6]. Azoospermia in KS is the consequence of progressive germ cells degeneration starting from mid-puberty [1], a critical period in which also Sertoli cells (SCs) function impairs in association with an extensive fibrosis and hyalinization of the seminiferous tubules and hyperplasia of interstitium and Leydig cells (LCs) [7]. The mechanisms leading to this global degeneration are still unclear, and no therapy is so far available for affected patients. A predominant hypothesis is that the altered dosage of genes escaping inactivation on the supernumerary X chromosome might affect the development and/or degeneration at meiosis of germ cells [7]. However, data about the role of X-expressed genes on testicular functions are inconsistent [8]. In this view, in recent years a number of studies have investigated gene expression profiles of normal and pathological testis, providing useful information about the molecular basis of the alteration of the spermatogenesis [5,9]. No study has previously investigated gene expression profiles in human KS testis. The present study is aimed to fill this gap, carrying out KS testicular gene expression profiling by a whole genome microarray approach, in order to provide global information about testis dysfunction, with particular regard to microenvironment alterations related to SC and LC deregulation.

## Methods

### Patients

The study was approved by the local Ethics Committee of the University Hospital of Padova and was in accordance with the Helsinki II Declaration. All participants were asked for and provided their informed consent. Bilateral testicular biopsies were obtained from 6 azoospermic non-mosaic KS patients (mean age 28.4 ± 3.7) affected by Sertoli cell-only syndrome and from 3 controls subjects (mean age 29.1 ± 4.4) with obstructive azoospermia and normal spermatogenesis. Details of method and processing of testicular biopsies are described in our previous study [9]. All KS patients had a full 47,XXY karyotype as evidenced by cytogenetic investigation on at least 50 metaphases, had not received testosterone substitution prior to biopsy, and had negative history for other possible causes of testicular damage. Controls were 3 subjects presenting with idiopathic obstructive azoospermia and histologically confirmed normal spermatogenesis. They had negative history, normal testes volumes and normal reproductive hormones (FSH, LH, total testosterone) levels. All KS and control men were Italian. Individual participants details which could allow identification were omitted to ensure anonymity.

### Microarray analysis

Testicular biopsies were homogenized using an hand glasspotter, and total RNA was extracted using the RNeasy® Microarray Tissue Mini Kit (Quiagen, Hilden, Germany). Total purified RNA was linearly amplified using the AminoAllylMessageAmp™ II aRNA Amplification Kit (Ambion, Austin, TX, USA). Ten μg of amplified aRNA were fluorescently labelled with Cy3 or Cy5 cyanins and then hybridized on the high-density arrays HOA_005 Human Whole Genome OneArray™ Microarray V5 (Phalanx Biotech, Belmont, CA, USA), containing 29,187 human probes. Each amplified KS testicular RNA was hybridized against an amplified RNA pool, obtained from the three non-pathological testis controls. Each array experiment was repeated as a dye-swap, for a total of 12 experiments. The Cy3/Cy5 fluorescent signals were captured by a Confocal Laser Scanner “ScanArrayExpress” (Packard BioScience, Meriden, CT, USA) and analysed using the software “ScanArrayExpress - MicroArray Analysis System” version 3.0 (Perkin Elmer, Waltham, MA, USA). The signal intensity medians from each spot were adjusted subtracting the local background intensity medians. Then, a LOWESS algorithm was used for raw data normalization. Array data were adjusted in order to remove ambiguous probes [10].

Raw data of the performed experiments have been recorded in the GEO public database (accession number: GSE54023).

### Statistical analysis

Genes were considered significantly expressed when showing an absolute fold change (FC) <0.7 or > 1.4, a present call in at least the 50% of experiments and a p-Value <0.05 (ANOVA). False Discovery Rate (FDR) was used to adjust p-values and to correct for the multiple testing issues [11]; FDR was set <10%. The resulting gene lists underwent a clustering analysis (Cluster 3.0 and TreeView, Stanford University Labs) to unravel Genes Differentially Expressed (DEGs), excluding transcripts showing a concordant value < 80%. Identified DEGs were, then, analysed by Ingenuity Pathways Analysis (IPA) software (Ingenuity Systems, Redwood City, CA, USA) to disclose the biological functions and the functional networks they are involved in.

### qRT-PCR microarray data validation

Microarray data were validated by quantitative Real-Time PCR (qRT-PCR) reaction, performed on the same RNA extracted from KS and controls testis biopsies, used for microarray analysis. Gene expression of 6 genes found significantly deregulated by microarray (HSD17B1, CAV1, SCARB1, ACVR2A, BRCA1, FANCD2) was evaluated. 2 μg of RNA were reverse transcribed using the High Capacity RNA-to-cDNA Kit (Applied Biosystems, UK). qRT-PCR was performed in a total volume of 30 μl containing 2x KAPA PROBE FAST ABI Prism qPCR kit (Kapa Biosystems, Wilmington, MA, USA) and 25 ng of cDNA using PrimeTime Mini qPCR assays (Integrated DNA technologies, Coralville, IA, USA) on an Abi 7900HT Sequencing Detection System (Applied Biosytems, UK). Specific primer and probe sets employed are available in Additional file 1: Table S5. Real time amplification conditions were: 10 minutes at 95°C followed by 40 cycles of 15 seconds at 95°C and 1 minute at 60°C. The selected genes relative expression was corrected against GAPDH and GUSB genes as endogenous controls. The ΔΔCt method was used to compare relative fold changes between samples and control. T-test was used to assess the p-Value, considering data significant when p < 0.05 [12].

## Results

**Figure 1:**
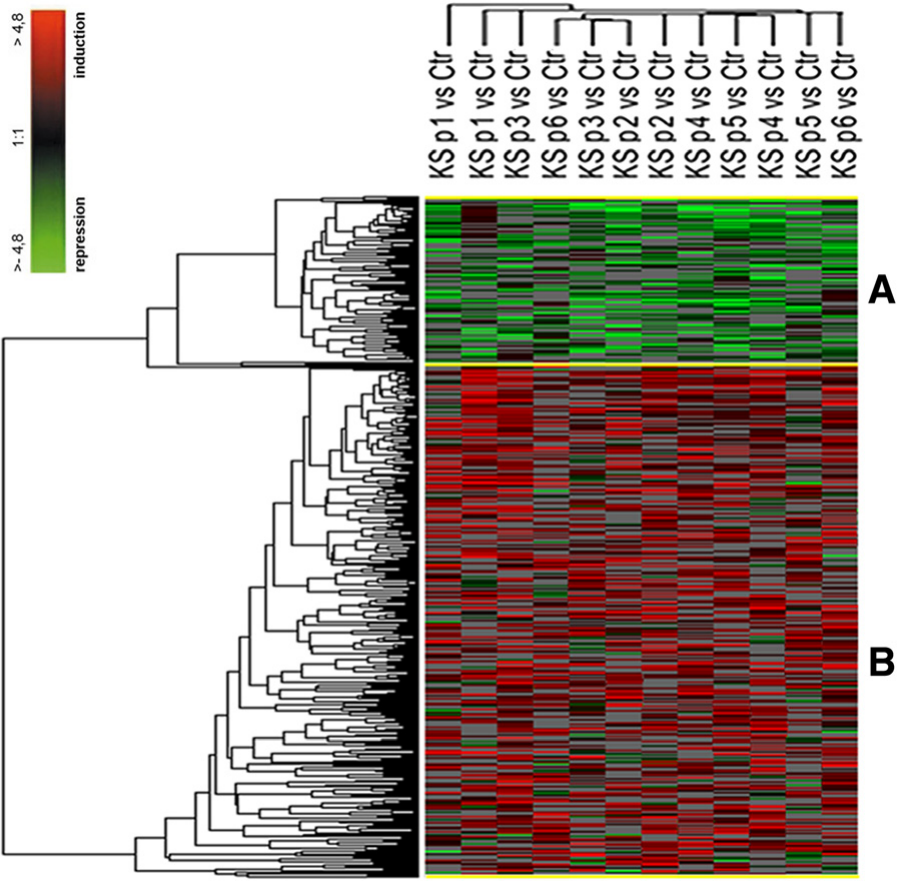
**Unsupervised hierarchical clustering analysis results.** The cluster analysis shows the presence of two different clusters composed respectively by 247 down-regulated genes (Cluster **A**) and 656 up-regulated transcripts (Cluster **B**). Each row represents a differentially expressed genes. In green the genes down-regulated, in red the genes up-regulated for each replicate. In grey and black are represented respectively the genes not modulated or with missing data for the given replicate. Each column represents an experimental condition, labelled as the specific KS patient vs. control. The columns labelled with the same name stand for the two performed replicate as dye-swap.

**Figure 2:**
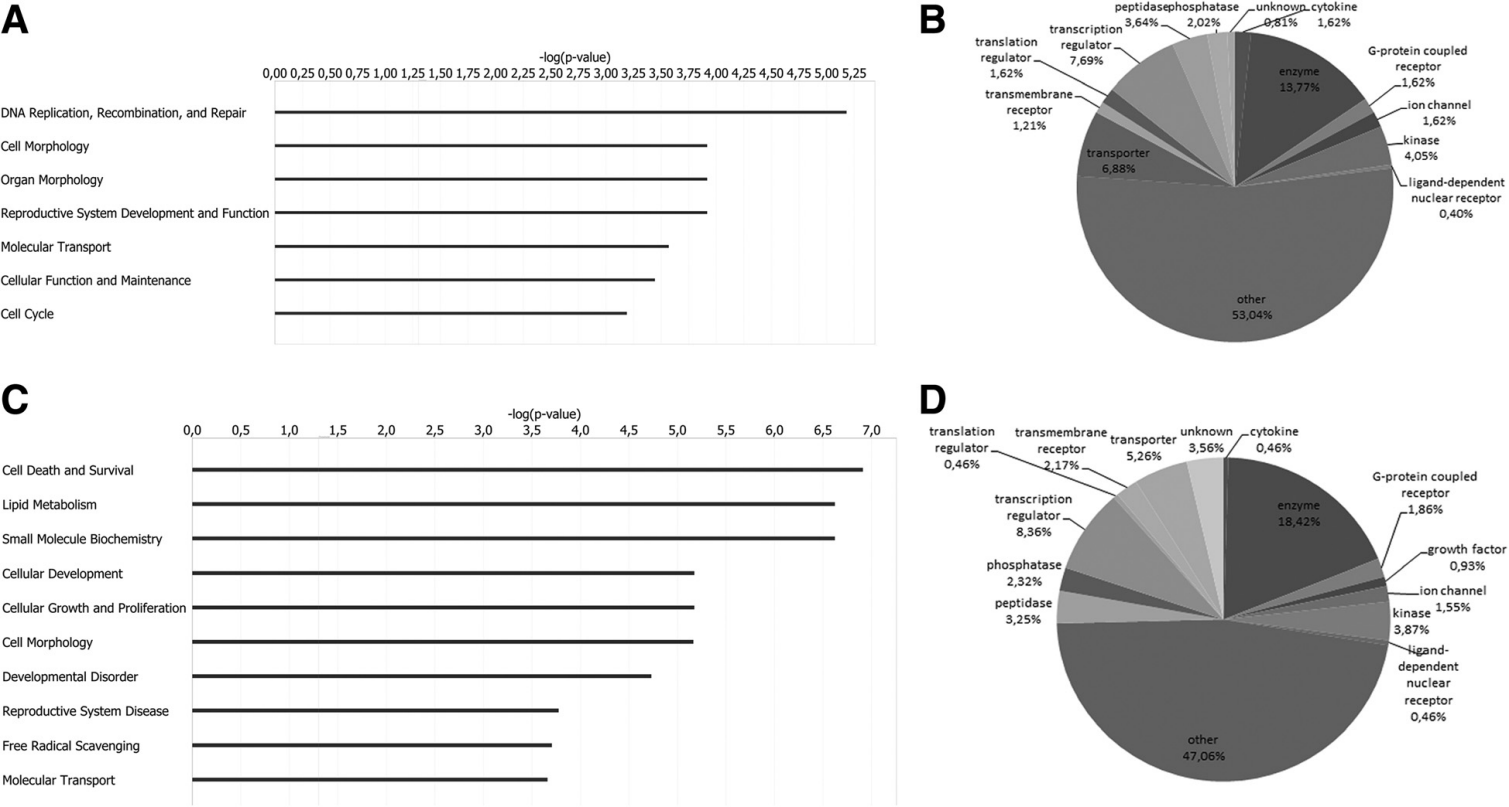
**IPA**-**inferred biological functions associated to Cluster A and B gene datasets. A)** Bar charts indicate IPA-inferred key biological functions modulated by down-regulated genes in Cluster **A**. **C)** Bar charts indicate IPA-inferred key biological functions modulated by up-regulated genes in Cluster **B**. The –log (p-value), is calculated by IPA based on the number of genes involved in the function and their reported role. **B**, **D)** Down and up-regulated transcripts in Cluster **A** and **B** classified for molecule type.

**Figure 3:**
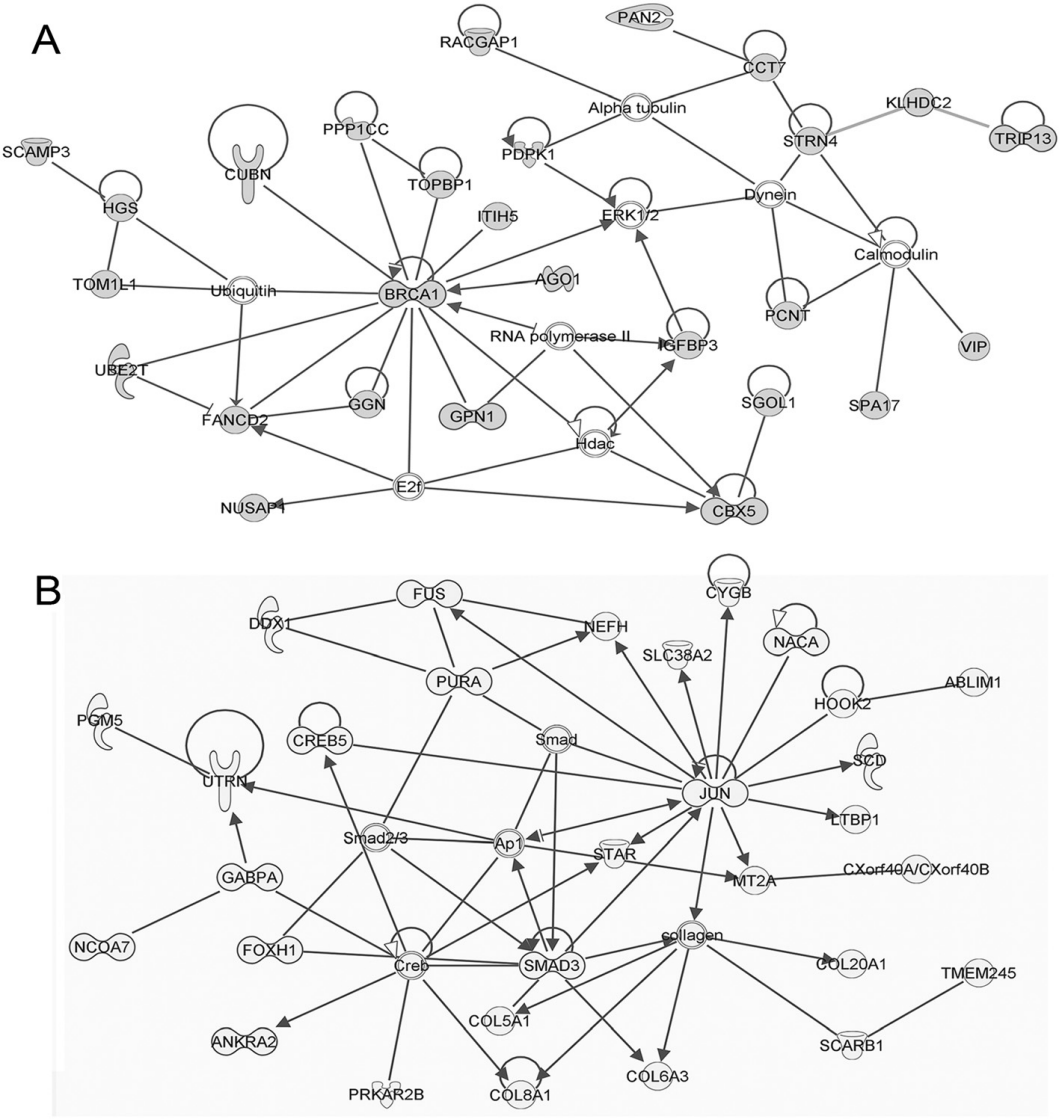
**IPA**-**inferred top networks associated to Cluster A and B gene datasets. A)** Top gene network generated by IPA for Cluster A down-regulated gene dataset. The network is centered around the key node gene BRCA1, involved in testis cell death. **B)** Top gene network generated by IPA for Cluster B up-regulated gene dataset. The central node of this network is SMAD3, involved in SCs functionality. Grey genes represent the deregulated genes associated to each Cluster while white genes represent those transcripts not modulated in KS testis vs. control testis.

**Figure 4:**
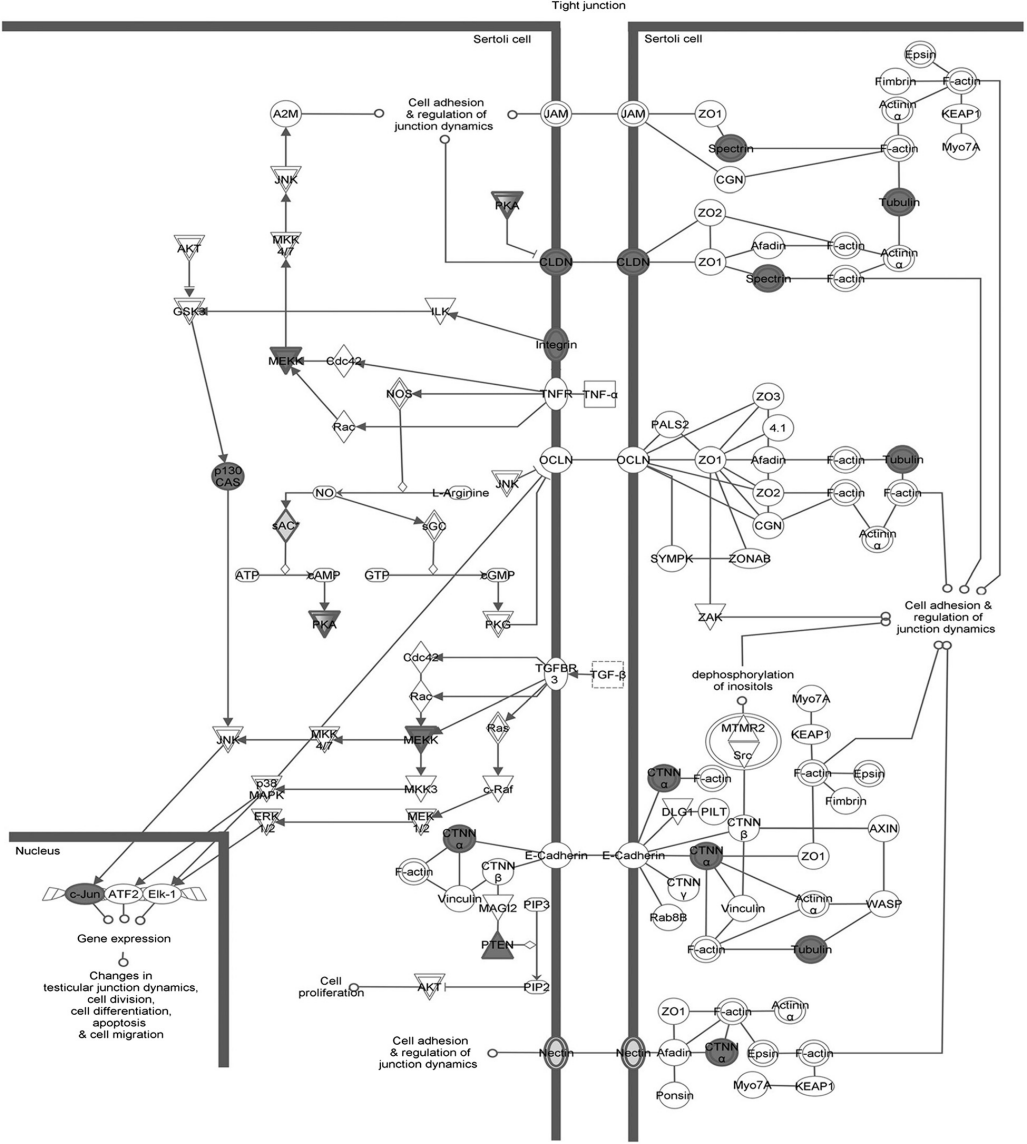
**IPA**-**inferred Sertoli cell** **–** **Sertoli cell junction signalling pathway.** The figure shows the significant deregulation of the Sertoli cell pathway generated by the analysis of both up- and down-regulated transcript datasets, mainly involved in Blood-Testis-Barrier maintenance. In grey are labelled the genes deregulated in KS testis vs. control testis.

Expression profiling analysis revealed the significant differential expression of 903 transcripts (Additional file 2: Table S1) in KS as compared to control testis. Two clusters of differentially expressed genes (Clusters A and B) were evidenced, composed by 247 down- and 656 over-regulated transcripts in KS testis (Figure 1, Additional file 3: Table S2 and Additional file 4: Table S3). IPA analysis revealed that the 247 down-expressed genes were mainly involved in the following biological functions: DNA replication, recombination and repair, cell morphology, organ morphology, reproductive system development and function, molecular transport, cellular function and maintenance, and cell cycle (Figure 2A, Additional file 4: Table S3). Among the down-expressed genes we found only 24 transcripts specifically expressed by the germinal epithelium, being the majority of under-regulated genes expressed by SCs and LCs. On the other hand, the 656 over-expressed genes resulted involved in the following biological functions: cell death and survival, lipid metabolism, small molecule biochemistry, cellular development, cellular growth and proliferation, cell morphology, developmental disorder, reproductive system disease, free radical scavenging and molecular transport (Figure 2B, Additional file 5: Table S4). Among these transcripts, we found only 1 up-regulated X-linked gene, namely SLC25A6, located in the PAR1, and 25 under-regulated genes specifically expressed in germ cells. IPA-inferred network analysis on the data set showed for cluster A 13 networks with a score ranging from 48 down to 15, and, for cluster B, 25 networks with a score ranging from 46 down to 15. The 2 top networks associated to each cluster are reported in Figure 3. IPA pathway analysis of the two clusters showed a significant deregulation of the Sertoli cell - Sertoli cell junction signalling pathway (Figure 4). When specifically focusing on the global function played by both down- and up-regulated genes, the following classes were evidenced:

- Testis cell death in KS and deregulation of genes involved in the control of apoptosis
  - proteins specifically modulating SCs and LCs survival and functionality (SMAD3, TNFRSF10B, TNFAIP3, BNIP3, BNIP3L, CCNG1, BRCA1, BTRC, FANCD2, PPP1C and TRIP13)
- Gene modulation of inflammatory response in KS testis
  - proteins involved in the inflammatory response (B2M, SOCS3, KRT6A, NTF4, TNFAIP3, IDE, PTEN, ZNF395, SYNE2, RAB34, GSN, INPP5D, LYPLA2, CTSL1, NR4A2, SELP, DUSP1, ICOS, PSAP, BNIP3L, ICOSGL).
- Alterations in the expression of genes involved in lipid metabolism, hormone synthesis and steroidogenesis
  - upstream regulators in hormones pathway (INHB, StAR, ANXA1, ANXA5)
  - steroidogenic enzymes (HSD17B1, HSD17B3, HSD17B4, HSD17B6, CYB5B, CYGB, GSTA2, MSMO1, SC5D, ACSL3, ASAH1, PTEN, TECR, AKR1C3, LIPA, NSMAF, SPTLC1, DLD, PCCA, SGPP1, ST8SIA1, SOD1, GABPA)
  - lipid transporters (SLC38A2, SLC16A12, SLC1A2, SLC2A1, APOH, GLTP, SCARB1, CAV1, CAV2, LDLR, RBP1, CLDN16, ABCD3)
- Genes involved in the regulation of blood-testis-barrier (BTB) show altered expression in KS testis
  - BTB formation and maintenance (BCAR1, CLDN5, CLDN16, CTNNA1, ITGA2, JUN, MAP3K6, MAP3K14, PRKAR1A, PRKAR2B, PTEN, SPTBN1, TUBA1A, ADCY10, PRKACG, PVRL3, CLU, FANCD2, CSNK2B)
- Down-regulation of genes specifically involved in sperm production
  - factors involved in spermatogenesis and male germ cells maturation and morphology (ACVR2A, TNP1, NME5, PPP1CC, TDRD1, and SPA17)

In order to validate the microarray results, quantitative Real-Time PCR (qRT-PCR) was performed on 3 genes in cluster A (ACVR2A, BRCA1 and FANCD2) and 3 genes in cluster B (HSD17B1, CAV1 and SCARB1). The relative expression of the selected genes was significant for each sample when compared to the expression in control testis (p < 0.05, Student’s T-test) (Additional file 6: Figure S1). qRT-PCR analysis confirmed microarray data showing no significant fold change difference in real time data when compared with microarray data (p > 0.05, Student’s T-test).

## Discussion

The analysis of the whole testis transcriptome in KS patients allowed us to identify the selective up- and down-expression of several genes involved in key molecular pathways related to KS testis global deregulation. A first important point was to analyse how many of the over-expressed genes mapped on the X chromosome, and thus to understand which portion of the up-regulation is due to the presence of an extra chromosome and which to the deregulation of testis gene expression.

Our study evidenced the up-regulation of a single X-linked gene, namely SLC25A6, mapped within the PAR1 region, suggesting that PAR regions genes have a minor role in testis functionality. Moreover, the lack of variation in X-linked genes out of PAR in our series confirms the well known inactivation of one X chromosome in KS patients [13], being the expression of X-linked genes restricted to spermatogonia [14], a cell type not present in our patients, all affected by azoospermia and showing a Sertoli cell-only condition. All these data together suggest that the KS extra X chromosome plays a central role in the early germ cell epithelium degeneration [14]. Thus, the large majority of over-expressed genes detected by our analysis reflect the deregulation induced by the 47,XXY condition in SCs and LCs. A similar picture can be depicted for down-regulated genes, among which we identified only few transcripts expressed in germinal epithelium, and thus under-expressed as a consequence of the lack of germ cells, and a larger number of transcripts whose under-regulation was the result of the alteration of the SCs and LCs transcriptome. Given these premises, we then focused our attention on transcripts involved in a series of testis functions, whose deregulation could explain the molecular basis of the testis impairment in these subjects. This analysis allowed us to identify a series of possible biological mechanisms involved in the pathogenesis of testicular dysfunction in KS, which are hereby discussed.

### Testis cell death in KS and deregulation of genes involved in the control of apoptosis

The presence of an increased apoptosis affecting SCs and LCs has been proposed as a main mechanism leading to KS testis dysfunction [7]. Our data support this hypothesis showing the over-expression of several genes responsible of an increased apoptotic process. SMAD3 was detected in this study as a central node in the top-network associated with the up-regulated gene dataset (Figure 3B). It is a key regulator of SCs differentiation and its over-expression can be associated with an impairment of SCs maturation process, reported as a feature of KS testis [15]. The increased cell death in KS testis can also be explained by the observed over-expression of TNFRSF10B, TNFAIP3, CCNG1, BNIP3 and BNIP3L known to regulate natural testes apoptosis and proliferative capacity of LCs [16-18]. Among the down-regulated transcripts we found genes that promote cell cycle progression (BRCA1, BTRC and TRIP13). The down-expression of these genes has been related to cell cycle block, apoptosis and reduced fertility in testis [19]. Interestingly, as shown in Figure 3A, BRCA1 is a key central node in the IPA top network associated with down-regulated genes dataset. All together these observations strongly suggest an active apoptotic process in KS testis.

### Gene modulation of inflammatory response in KS testis

IPA analysis of cluster B dataset evidenced that several pro-apoptotic genes are also involved in the inflammatory response. In particular, B2M has been described to be expressed by SCs and its over-expression has been previously reported to induce antigen presentation and apoptosis in SCs, thus deregulating testis immune-privileged microenvironment [20]. The disruption of the immune-privileged microenvironment is also suggested by the over-expression of ICOSLG gene involved in the activation and regulation of the adaptive immune responses in a number of tissue as well as the pathological testis [21]. On the other hand, our study evidenced an over-expression of SOCS3 that acts as a negative regulator of inflammatory response and its up-regulation suggests the development of protective mechanisms in response to the severe inflammation that seems to affect KS testes [22]. Taken together, these data suggest that deregulation of genes involved in the inflammation process might be responsible of some features of testicular histological findings in KS, especially the high degree of fibrosis that is invariably described as an early event in the testicular involution process of these subjects [7].

### Alterations in the expression of genes involved in lipid metabolism, hormone synthesis and steroidogenesis

KS patients are described as hypergonadotropic hypogonadal subjects with invariably high LH and FSH serum levels as a consequence of low levels of circulating testosterone and inhibin B, produced by LCs and SCs, respectively [23]. The analysis of the up-regulated transcripts in our series revealed 97 genes involved in lipid metabolism, the majority of which specifically coding for factors involved in hormones pathways and steroidogenesis. Among these, INHBA subunity encodes for the major regulators of FSH secretion in the adult male. The detected over-regulation may indicate an over production of activin (homodimer formed by two INHBA subunits) [24] which could explain the over secretion of FSH reported in KS patients, or the hyperstimulation of SCs through higher FSH levels together with altered Inhibin B release into the bloodstream, as suggested for testosterone produced by LCs [25]. ANXA1 and ANXA5 are important mediator of the hypotalamo-pitutitary axis, regulating testosterone production. The effect of their over-expression, observed in KS testis, could be related to LCs dysfunction resulting in LCs death [26,27]. StAR, SCARB1, CAV1 and CAV2 mediate the traffiking of cholesterol, essential to begin the synthesis of pregnenolone. The observed over-expression of these genes likely might reflect the hyperactivation of the steroidogenic activity of the LCs [28,29]. Supporting this hypothesis, it has been recently demonstrated that intratesticular testosterone concentrations in KS are higher than normal, and the low testosterone serum levels commonly seen in this syndrome could be related to altered released into the bloodstream [25]. Accordingly to this latter hypothesis, we found also up-regulation of HSD17B1 that encodes for the enzyme that converts androstenedione in testosterone during steroidogenesis. These findings might well be explained by the high levels of LH that stimulate LC hyperplasia and hyperfunction. Taken together, these unexpected data suggest an over activation of the hormones biosynthetic pathway in SCs and LCs, in contrast with the common view that KS subjects are hypogonadal because of a LC dysfunction that renders them unresponsive to LH, but completely in agreement with more recent findings [25].

### Genes involved in the regulation of blood-testis-barrier (BTB) show altered expression in KS testis

BTB is a unique structure characterized by different types of junctions between SCs, that ensure a strictly controlled environment for post-meiotic germ cells, and is regulated by several paracrine and autocrine factors [30]. Perturbations of the BTB are associated with spermatogenesis failure and testicular microenvironment deregulation [31]. We found altered expression of several genes in the Sertoli cell Junction Signaling pathway (Figure 4). Among genes present in the pathway, CLDN5 and CLDN16 (up-regulated), FANCD2 and CLU (down-regulated) are of particular interest because they code for key proteins involved in BTB structuring. CLDN5 and CLDN16 encode for components of tight junction strands and their over-expression could reflect either a protective mechanism of response against the testicular microenvironment disruption or inflammation response, or the presence of immature SCs [32]. FANCD2 product regulates in testis the seminiferous tubules maintenance, and its down-expression has been associated with reduction of germ cells number [33]. CLU is synthesized by SCs and regulates the germ cells proliferation and differentiation and its down-expression could be the consequence of germ cells absence. Among the down-regulated transcripts, there are also 30 genes that regulate molecular transport producing in KS testis a BTB barrier unable to regulate this central process [34]. Finally, IPA analysis showed the up-regulation of several collagens transcripts (COL20A1, COL5A1, COL6A3, COL8A1) participating to the fibrosis and hyalinization processes, well known features of KS testis [1]. The over regulation of collagens is reported also to ‘de-stabilizes’ the BTB, via an interaction with laminin fragments [35]. In this view, it is interesting to note that among up-regulated transcipts there are also two genes encoding for laminins (LAMB3 and LAMC3). All together, these findings suggest a compromised BTB and support the well-known presence of fibrosis in the testis of KS, indicating another mechanism possibly involved in the spermatogenic impairment of these subjects.

### Down-regulation of genes specifically involved in sperm production

IPA analysis of Cluster A gene dataset confirmed that, when compared to testis with normal spermatogenesis, KS testis under-expresses central genes involved in spermatogenesis and male germ cells maturation and morphology [1,36,37]. Among these, ACVR2A and TNP1 are transcripts of particular interest. ACVR2A is a member of the TGF-beta superfamily that regulates cell development and maturation. Its expression was described to be central in germ cells and SCs maturation and its reduced level has been linked to germ cells and SCs proliferation block [38]. TNP1 intermediates the histones replacement by protamines, being a marker of early spermatogenesis [39] and consequently its reduced level indicates germ cells maturation defects.

## Conclusions

In conclusion, the increased LCs steroidogenic function together with the impairment of inflammatory pathways and BTB structure, result in increased apoptosis leading to the pathological features of KS testis (fibrosis and hyalinization of seminiferous tubules, LC hyperplasia). Furthermore, according to the testicular microenvironment hypothesis, our findings indicate that the decline of spermatogenesis give rise to an high degree of SCs and LCs dysfunctions and thus the impaired ability to provide an adequate microenvironment that supports germ cells development [40]. These transcriptome data could serve as the basis for future functional studies to fully understand the molecular mechanisms leading to KS testis global deregulation.

### Future perspectives

Further studies on gene expression profile in younger Klinefelter subjects could be interesting to disclose the causes leading to the degeneration of the cross-talk between different cellular ultrastructures in the seminiferous epithelium unable to coordinate the different spermatogenesis stages leading to the degeneration of the germ cells in the KS adult. These information, associated with an early diagnosis could help to unravel possible therapeutic targets for testis failure prevention and limitation.

### Availability of supporting data

The data sets supporting the results of this article are available in the GEO public repository database, [ID: GSE54023; http://www.ncbi.nlm.nih.gov/geo/query/acc.cgi?acc=GSE54023].

## Additional files

Additional file 1: Table S5. qRT-PCR primers and probes set. Additional file 2: Table S1. Alphabetical list of the 247 down-expressed genes in Cluster A. Additional file 3: Table S2. Alphabetical list of the 656 up-expressed genes in Cluster B. Additional file 4: Table S3. IPA functional analysis of cluster A transcripts. Additional file 5: Table S4. IPA functional analysis of cluster B transcripts. Additional file 6: Figure S1. Validation of microarray gene expression data by qRT-PCR. Bar graph shows mRNA levels of HSD17B1, CAV1, SCARB1, ACVR2A, BRCA1, FANCD2 as measured by quantitative real time PCR in each of KS testis analysed vs. Control testis. qRT-PCR data are expressed as mean values of relative fold changes ± SD of three independent experiments performed in triplicate. qRT-PCR data, for each gene, were significant (p<0.05, Student’s T-test) when compared to Control testis. qRT-PCR data did not show significant difference when compared to MicroArray data (p>0.05, Student’s T-test).
