# Supplementary material for: Deregulation of sertoli and leydig cells function in patients with klinefelter syndrome as evidenced by testis transcriptome analysis
Source: BMC Genomics. 2015 Mar 7;16(1):156. doi: 10.1186/s12864-015-1356-0 (PMC4362638; doi:10.1186/s12864-015-1356-0)
Supplement: Additional file 1: Table S5. — qRT-PCR primers and probes set. [file 12864_2015_1356_MOESM1_ESM.docx]

Table S5: qRT-PCR primers and probes set

| GENE ID | Forward Primer | Reverse Primer | Probe |
| --- | --- | --- | --- |
| HSD17B1 | 5'-GAAGGCTTATGCGAGAGTCTG-3' | 5'-GAAGGTGTGGATGTCCGTG-3' | 5'-/56-FAM/TCATGGAGA/ZEN/AGGTGTTGGGCAGC/3IABkFQ/-3' |
| CAV1 | 5'-CCTTCCTCAGTTCCCTTAAAGC-3' | 5'-TGTAGATGTTGCCCTGTTCC-3' | 5'-/56-FAM/TCCTCACAG/ZEN/TTTTCATCCAGCCACG/3IABkFQ/-3' |
| SCARB1 | 5'-ATCCTCACTTCCTCAACGC-3' | 5'-TTCACAGAGCAGTTCATGGG-3' | 5'-/56-FAM/TGACTGGCC/ZEN/TGCACCCTAACC/3IABkFQ/-3' |
| ACVR2A | 5'-CCTGGAATGAAGCATGAGAAC-3' | 5'-TTCCAAGAGACCACATTAGCC-3' | 5'-/56-FAM/AGCCAAAGA/ZEN/TCCACATCAACACTGGT/3IABkFQ/-3' |
| BRCA1 | 5'-GCCTTCTAACAGCTACCCTC-3' | 5'-CTTCTGGATTCTGGCTTATAGGG-3' | 5'-/56-FAM/TTTGTTCTG/ZEN/GATTTCGCAGGTCCTCA/3IABkFQ/-3' |
| FANCD2 | 5'-GGAGTCCATGTCTGCTAAAGAG-3' | 5'-CAATGTCTTTAACCGAGTGAG-3' | 5'-/56-FAM/AGGTGATGT/ZEN/TTCCTGGCAGAAGGC/3IABkFQ/-3' |
| GAPDH | 5'-CTTTGTCAAGCTCATTTCCTGG-3' | 5'-TCTTGCTCAGTGTCCTTGC-3' | 5'-/56-FAM/CACCCTGTT/ZEN/GCTGTAGCCGTATTCA/3IABkFQ/-3' |
| GUSB | 5'-AGGTGATGGAAGAAGTGGTG-3' | 5'-AGGATTTGGTGTGAGCGATC-3' | 5'-/56-FAM/CAGATTCTA/ZEN/GGTGGGACGCAGGC/3IABkFQ/-3' |
