# Supplementary material for: Deregulation of sertoli and leydig cells function in patients with klinefelter syndrome as evidenced by testis transcriptome analysis
Source: BMC Genomics. 2015 Mar 7;16(1):156. doi: 10.1186/s12864-015-1356-0 (PMC4362638; doi:10.1186/s12864-015-1356-0)
Supplement: Additional file 2: Table S1. — Alphabetical list of the 247 down-expressed genes in Cluster A. [file 12864_2015_1356_MOESM2_ESM.docx]

Table S1: alphabetical list of the 247 down-expressed genes in Cluster A

|  | **ID** | **SYMBOL** | **ENTREZ GENE NAME** | **LOCATION** | **TYPE(S)** |
| --- | --- | --- | --- | --- | --- |
| **1** | NM_001025357.2 | AARD | alanine and arginine rich domain containing protein | Other | other |
| **2** | NM_007188.3 | ABCB8 | ATP-binding cassette, sub-family B (MDR/TAP), member 8 | Cytoplasm | transporter |
| **3** | NM_014049.4 | ACAD9 | acyl-CoA dehydrogenase family, member 9 | Cytoplasm | enzyme |
| **4** | NM_005736.3 | ACTR1A | ARP1 actin-related protein 1 homolog A, centractin alpha (yeast) | Cytoplasm | other |
| **5** | NM_001616.3 | ACVR2A | activin A receptor, type IIA | Plasma Membrane | kinase |
| **6** | NM_001167749.1 | ADCY10 | adenylate cyclase 10 (soluble) | Cytoplasm | enzyme |
| **7** | NM_001167749.1 | ADCY10 | adenylate cyclase 10 (soluble) | Cytoplasm | enzyme |
| **8** | NM_002285.2 | AFF3 | AF4/FMR2 family, member 3 | Nucleus | transcription regulator |
| **9** | NM_012199.2 | AGO1 | argonaute RISC catalytic component 1 | Cytoplasm | translation regulator |
| **10** | NM_001001891.3 | ANO7 | anoctamin 7 | Plasma Membrane | ion channel |
| **11** | NM_006051.3 | APBB3 | amyloid beta (A4) precursor protein-binding, family B, member 3 | Cytoplasm | other |
| **12** | NM_004311.2 | ARL3 | ADP-ribosylation factor-like 3 | Cytoplasm | enzyme |
| **13** | NM_001031739.2 | ASB9 | ankyrin repeat and SOCS box containing 9 | Nucleus | transcription regulator |
| **14** | NM_024085.3 | ATG9A | autophagy related 9A | Cytoplasm | other |
| **15** | NM_001697.2 | ATP5O | ATP synthase, H+ transporting, mitochondrial F1 complex, O subunit | Cytoplasm | transporter |
| **16** | NM_004231.2 | ATP6V1F | ATPase, H+ transporting, lysosomal 14kDa, V1 subunit F | Other | enzyme |
| **17** | NM_144696.4 | AXDND1 | axonemal dynein light chain domain containing 1 | Extracellular Space | other |
| **18** | NM_178537.4 | B4GALNT4 | beta-1,4-N-acetyl-galactosaminyl transferase 4 | Extracellular Space | enzyme |
| **19** | NM_203314.2 | BDH1 | 3-hydroxybutyrate dehydrogenase, type 1 | Cytoplasm | enzyme |
| **20** | NR_027676.1 | BRCA1 | breast cancer 1, early onset | Nucleus | transcription regulator |
| **21** | NM_033637.2 | BTRC | beta-transducin repeat containing E3 ubiquitin protein ligase | Cytoplasm | enzyme |
| **22** | NM_001164375.2 | C10orf105 | chromosome 10 open reading frame 105 | Other | other |
| **23** | NM_207645.3 | C11orf87 | chromosome 11 open reading frame 87 | Other | other |
| **24** | NM_207430.2 | C11orf88 | chromosome 11 open reading frame 88 | Other | other |
| **25** | NM_032413.2 | C15orf48 | chromosome 15 open reading frame 48 | Nucleus | other |
| **26** | NM_032561.2 | C22orf23 | chromosome 22 open reading frame 23 | Other | other |
| **27** | NM_152786.1 | C9orf43 | chromosome 9 open reading frame 43 | Other | other |
| **28** | NM_004291.3 | CARTPT | CART prepropeptide | Extracellular Space | other |
| **29** | NM_005093.3 | CBFA2T2 | core-binding factor, runt domain, alpha subunit 2; translocated to, 2 | Nucleus | transcription regulator |
| **30** | NM_001085457.1 | CBWD3/CBWD6 | COBW domain containing 6 | Other | other |
| **31** | NM_012117.2 | CBX5 | chromobox homolog 5 | Nucleus | transcription regulator |
| **32** | NM_152775.3 | CCDC110 | coiled-coil domain containing 110 | Nucleus | other |
| **33** | NM_024553.2 | CCDC132 | coiled-coil domain containing 132 | Other | other |
| **34** | NM_022757.4 | CCDC14 | coiled-coil domain containing 14 | Cytoplasm | other |
| **35** | NM_020879.2 | CCDC146 | coiled-coil domain containing 146 | Other | other |
| **36** | NM_001166284.1 | CCT7 | chaperonin containing TCP1, subunit 7 (eta) | Cytoplasm | other |
| **37** | NM_080668.3 | CDCA5 | cell division cycle associated 5 | Cytoplasm | other |
| **38** | NM_002768.2 | CHMP1A | charged multivesicular body protein 1A | Extracellular Space | peptidase |
| **39** | NM_000747.2 | CHRNB1 | cholinergic receptor, nicotinic, beta 1 (muscle) | Plasma Membrane | transmembrane receptor |
| **40** | NM_001040138.1 | CKLF | chemokine-like factor | Extracellular Space | cytokine |
| **41** | NM_016326.2 | CKLF | chemokine-like factor | Extracellular Space | cytokine |
| **42** | NM_001171138.1 | CLU | clusterin | Cytoplasm | other |
| **43** | NM_020188.3 | CMC2 | COX assembly mitochondrial protein 2 homolog (S. cerevisiae) | Cytoplasm | other |
| **44** | NM_080680.2 | COL11A2 | collagen, type XI, alpha 2 | Extracellular Space | other |
| **45** | NM_080538.2 | COLQ | collagen-like tail subunit (single strand of homotrimer) of asymmetric acetylcholinesterase | Extracellular Space | other |
| **46** | NM_004236.3 | COPS2 | COP9 signalosome subunit 2 | Cytoplasm | transcription regulator |
| **47** | NM_031476.3 | CRISPLD2 | cysteine-rich secretory protein LCCL domain containing 2 | Cytoplasm | other |
| **48** | NM_001320.5 | CSNK2B | casein kinase 2, beta polypeptide | Cytoplasm | kinase |
| **49** | NM_001143775.1 | CTDNEP1 | CTD nuclear envelope phosphatase 1 | Extracellular Space | phosphatase |
| **50** | NM_001081.3 | CUBN | cubilin (intrinsic factor-cobalamin receptor) | Plasma Membrane | transmembrane receptor |
| **51** | NM_003591.2 | CUL2 | cullin 2 | Nucleus | enzyme |
| **52** | NM_002996.3 | CX3CL1 | chemokine (C-X3-C motif) ligand 1 | Extracellular Space | cytokine |
| **53** | NM_001916.3 | CYC1 | cytochrome c-1 | Cytoplasm | enzyme |
| **54** | NM_015533.3 | DAK | dihydroxyacetone kinase 2 homolog (S. cerevisiae) | Cytoplasm | other |
| **55** | NM_016286.2 | DCXR | dicarbonyl/L-xylulose reductase | Other | enzyme |
| **56** | NM_020664.3 | DECR2 | 2,4-dienoyl CoA reductase 2, peroxisomal | Cytoplasm | enzyme |
| **57** | NM_023036.4 | DNAI2 | dynein, axonemal, intermediate chain 2 | Cytoplasm | other |
| **58** | NM_032364.5 | DNAJC14 | DnaJ (Hsp40) homolog, subfamily C, member 14 | Cytoplasm | other |
| **59** | NM_012100.2 | DNPEP | aspartyl aminopeptidase | Cytoplasm | peptidase |
| **60** | NM_177991.1 | DUSP15 | dual specificity phosphatase 15 | Cytoplasm | phosphatase |
| **61** | NM_001008394.1 | EID3 | EP300 interacting inhibitor of differentiation 3 | Cytoplasm | other |
| **62** | NM_003756.2 | EIF3H | eukaryotic translation initiation factor 3, subunit H | Cytoplasm | translation regulator |
| **63** | NM_002212.2 | EIF6 | eukaryotic translation initiation factor 6 | Cytoplasm | translation regulator |
| **64** | NM_001008493.1 | ENAH | enabled homolog (Drosophila) | Plasma Membrane | other |
| **65** | NM_152512.3 | ENTHD1 | ENTH domain containing 1 | Other | other |
| **66** | NM_001099439.1 | EPHA10 | EPH receptor A10 | Plasma Membrane | transmembrane receptor |
| **67** | NM_006544.3 | EXOC5 | exocyst complex component 5 | Cytoplasm | other |
| **68** | NR_024572.1 | FAM213A | family with sequence similarity 213, member A | Extracellular Space | other |
| **69** | NM_033084.3 | FANCD2 | Fanconi anemia, complementation group D2 | Nucleus | other |
| **70** | NM_145235.3 | FANK1 | fibronectin type III and ankyrin repeat domains 1 | Nucleus | transcription regulator |
| **71** | NM_033085.2 | FATE1 | fetal and adult testis expressed 1 | Cytoplasm | other |
| **72** | NM_002011.3 | FGFR4 | fibroblast growth factor receptor 4 | Plasma Membrane | kinase |
| **73** | NM_002031.2 | FRK | fyn-related kinase | Nucleus | kinase |
| **74** | NM_032135.3 | FSCB | fibrous sheath CABYR binding protein | Plasma Membrane | other |
| **75** | NM_007278.1 | GABARAP | GABA(A) receptor-associated protein | Cytoplasm | transporter |
| **76** | NM_001127621.1 | GALE | UDP-galactose-4-epimerase | Cytoplasm | enzyme |
| **77** | NM_152657.3 | GGN | gametogenetin | Nucleus | other |
| **78** | NM_032569.3 | GLYR1 | glyoxylate reductase 1 homolog (Arabidopsis) | Other | other |
| **79** | NM_052847.2 | GNG7 | guanine nucleotide binding protein (G protein), gamma 7 | Plasma Membrane | enzyme |
| **80** | NM_004871.2 | GOSR1 | golgi SNAP receptor complex member 1 | Cytoplasm | transporter |
| **81** | NR_026735.1 | GPN1 | GPN-loop GTPase 1 | Nucleus | transcription regulator |
| **82** | NM_005301.2 | GPR35 | G protein-coupled receptor 35 | Plasma Membrane | G-protein coupled receptor |
| **83** | NM_017577.4 | GRAMD1C | GRAM domain containing 1C | Other | other |
| **84** | NM_001077244.1 | GRIA4 | glutamate receptor, ionotropic, AMPA 4 | Plasma Membrane | ion channel |
| **85** | NM_005326.4 | HAGH | hydroxyacylglutathione hydrolase | Cytoplasm | enzyme |
| **86** | NM_024511.5 | HAUS3 | HAUS augmin-like complex, subunit 3 | Cytoplasm | other |
| **87** | NM_001145207.1 | HBS1L | HBS1-like (S. cerevisiae) | Cytoplasm | translation regulator |
| **88** | NM_004712.3 | HGS | hepatocyte growth factor-regulated tyrosine kinase substrate | Cytoplasm | other |
| **89** | NM_001098786.1 | HILPDA | hypoxia inducible lipid droplet-associated | Cytoplasm | other |
| **90** | NM_016287.3 | HP1BP3 | heterochromatin protein 1, binding protein 3 | Nucleus | other |
| **91** | NM_001013398.1 | IGFBP3 | insulin-like growth factor binding protein 3 | Extracellular Space | other |
| **92** | NM_033439.2 | IL33 | interleukin 33 | Extracellular Space | cytokine |
| **93** | NM_172374.1 | IL4I1 | interleukin 4 induced 1 | Cytoplasm | enzyme |
| **94** | NM_004515.2 | ILF2 | interleukin enhancer binding factor 2 | Nucleus | transcription regulator |
| **95** | NM_033547.3 | INTS4 | integrator complex subunit 4 | Nucleus | other |
| **96** | NM_032817.4 | ITIH5 | inter-alpha-trypsin inhibitor heavy chain family, member 5 | Other | other |
| **97** | NM_006766.3 | KAT6A | K(lysine) acetyltransferase 6A | Nucleus | enzyme |
| **98** | NM_018240.5 | KIRREL | kin of IRRE like (Drosophila) | Plasma Membrane | other |
| **99** | NM_014315.2 | KLHDC2 | kelch domain containing 2 | Cytoplasm | other |
| **100** | NM_001025231.1 | KPRP | keratinocyte proline-rich protein | Other | other |
| **101** | NM_005560.3 | LAMA5 | laminin, alpha 5 | Extracellular Space | other |
| **102** | NM_197958.1 | LARP6 | La ribonucleoprotein domain family, member 6 | Other | other |
| **103** | NM_001010978.2 | LDLRAD1 | low density lipoprotein receptor class A domain containing 1 | Other | other |
| **104** | NR_027284.1 | LINC00602 | long intergenic non-protein coding RNA 602 | Other | other |
| **105** | NR_027254.1 | LINC00671 | long intergenic non-protein coding RNA 671 | Other | other |
| **106** | NR_026899.1 | LOC146880 | Rho GTPase activating protein 27 pseudogene | Other | other |
| **107** | NM_001102659.1 | LOC200726 | hCG1657980 | Other | other |
| **108** | NM_001162371.1 | LOC728392 | uncharacterized LOC728392 | Other | other |
| **109** | NM_002319.3 | LRCH4 | leucine-rich repeats and calponin homology (CH) domain containing 4 | Cytoplasm | transcription regulator |
| **110** | NM_024036.4 | LRFN4 | leucine rich repeat and fibronectin type III domain containing 4 | Other | other |
| **111** | NM_012321.3 | LSM4 | LSM4 homolog, U6 small nuclear RNA associated (S. cerevisiae) | Nucleus | other |
| **112** | NM_033364.3 | MAATS1 | MYCBP-associated, testis expressed 1 | Cytoplasm | other |
| **113** | NM_022149.4 | MAGEF1 | melanoma antigen family F, 1 | Other | other |
| **114** | NM_019090.2 | MAP10 | microtubule-associated protein 10 | Extracellular Space | other |
| **115** | NM_018067.3 | MAP7D1 | MAP7 domain containing 1 | Other | other |
| **116** | NM_015112.2 | MAST2 | microtubule associated serine/threonine kinase 2 | Cytoplasm | kinase |
| **117** | NM_133486.2 | MBNL3 | muscleblind-like splicing regulator 3 | Nucleus | other |
| **118** | NM_032793.3 | MFSD2A | major facilitator superfamily domain containing 2A | Other | other |
| **119** | NM_018944.2 | MIS18A | MIS18 kinetochore protein homolog A (S. pombe) | Nucleus | other |
| **120** | NM_005439.2 | MLF2 | myeloid leukemia factor 2 | Nucleus | other |
| **121** | XM_293325.7 | MPC1L | mitochondrial pyruvate carrier 1-like | Other | other |
| **122** | NM_032111.2 | MRPL14 | mitochondrial ribosomal protein L14 | Cytoplasm | other |
| **123** | NM_001098835.1 | MS4A15 | membrane-spanning 4-domains, subfamily A, member 15 | Other | other |
| **124** | NM_170721.1 | MSI2 | musashi RNA-binding protein 2 | Cytoplasm | other |
| **125** | NM_025029.3 | MZT2B | mitotic spindle organizing protein 2B | Cytoplasm | other |
| **126** | NM_001113201.1 | NACA | nascent polypeptide-associated complex alpha subunit | Cytoplasm | transcription regulator |
| **127** | NM_015909.2 | NBAS | neuroblastoma amplified sequence | Other | other |
| **128** | NM_001010980.4 | NCMAP | noncompact myelin associated protein | Plasma Membrane | other |
| **129** | NM_004551.2 | NDUFS3 | NADH dehydrogenase (ubiquinone) Fe-S protein 3, 30kDa (NADH-coenzyme Q reductase) | Cytoplasm | enzyme |
| **130** | NM_172390.1 | NFATC1 | nuclear factor of activated T-cells, cytoplasmic, calcineurin-dependent 1 | Nucleus | transcription regulator |
| **131** | NM_134444.4 | NLRP4 | NLR family, pyrin domain containing 4 | Other | other |
| **132** | NM_003551.2 | NME5 | NME/NM23 family member 5 | Other | kinase |
| **133** | NM_024313.2 | NOL12 | nucleolar protein 12 | Nucleus | other |
| **134** | NM_003298.3 | NR2C2 | nuclear receptor subfamily 2, group C, member 2 | Nucleus | ligand-dependent nuclear receptor |
| **135** | NM_018454.6 | NUSAP1 | nucleolar and spindle associated protein 1 | Nucleus | other |
| **136** | NM_001004724.1 | OR4N5 | olfactory receptor, family 4, subfamily N, member 5 | Plasma Membrane | G-protein coupled receptor |
| **137** | NM_001145373.2 | OTUD1 | OTU domain containing 1 | Other | other |
| **138** | NM_138381.2 | OXNAD1 | oxidoreductase NAD-binding domain containing 1 | Cytoplasm | transporter |
| **139** | NM_000437.3 | PAFAH2 | platelet-activating factor acetylhydrolase 2, 40kDa | Cytoplasm | enzyme |
| **140** | NM_001166279.1 | PAN2 | PAN2 poly(A) specific ribonuclease subunit homolog (S. cerevisiae) | Cytoplasm | peptidase |
| **141** | NM_006437.3 | PARP4 | poly (ADP-ribose) polymerase family, member 4 | Other | enzyme |
| **142** | NM_018386.2 | PCID2 | PCI domain containing 2 | Nucleus | transcription regulator |
| **143** | NM_006031.5 | PCNT | pericentrin | Cytoplasm | other |
| **144** | NM_182612.2 | PDDC1 | Parkinson disease 7 domain containing 1 | Other | other |
| **145** | NM_002603.2 | PDE7A | phosphodiesterase 7A | Cytoplasm | enzyme |
| **146** | NM_002613.3 | PDPK1 | 3-phosphoinositide dependent protein kinase-1 | Cytoplasm | kinase |
| **147** | NM_178140.2 | PDZD2 | PDZ domain containing 2 | Plasma Membrane | other |
| **148** | NM_153812.2 | PHF13 | PHD finger protein 13 | Nucleus | other |
| **149** | NM_015020.2 | PHLPP2 | PH domain and leucine rich repeat protein phosphatase 2 | Cytoplasm | enzyme |
| **150** | NM_012407.3 | PICK1 | protein interacting with PRKCA 1 | Cytoplasm | enzyme |
| **151** | NR_023916.1 | PIN1P1 | peptidylprolyl cis/trans isomerase, NIMA-interacting 1 pseudogene 1 | Other | other |
| **152** | NM_001130964.1 | PLCD1 | phospholipase C, delta 1 | Cytoplasm | enzyme |
| **153** | NM_025201.4 | PLEKHO2 | pleckstrin homology domain containing, family O member 2 | Cytoplasm | other |
| **154** | NR_024593.1 | POM121L10P | POM121 transmembrane nucleoporin-like 10, pseudogene | Other | other |
| **155** | NR_027694.1 | PP2D1 | protein phosphatase 2C-like domain containing 1 | Other | phosphatase |
| **156** | NM_002710.2 | PPP1CC | protein phosphatase 1, catalytic subunit, gamma isozyme | Cytoplasm | phosphatase |
| **157** | NR_002191.2 | PPP1R2P9 | protein phosphatase 1, regulatory (inhibitor) subunit 2 pseudogene 9 | Other | other |
| **158** | NM_002732.3 | PRKACG | protein kinase, cAMP-dependent, catalytic, gamma | Cytoplasm | kinase |
| **159** | NM_002789.4 | PSMA4 | proteasome (prosome, macropain) subunit, alpha type, 4 | Cytoplasm | peptidase |
| **160** | NM_002794.3 | PSMB2 | proteasome (prosome, macropain) subunit, beta type, 2 | Cytoplasm | peptidase |
| **161** | NM_005805.4 | PSMD14 | proteasome (prosome, macropain) 26S subunit, non-ATPase, 14 | Cytoplasm | peptidase |
| **162** | NM_000955.2 | PTGER1 | prostaglandin E receptor 1 (subtype EP1), 42kDa | Plasma Membrane | G-protein coupled receptor |
| **163** | NM_012232.5 | PTRF | polymerase I and transcript release factor | Nucleus | transcription regulator |
| **164** | NM_015480.1 | PVRL3 | poliovirus receptor-related 3 | Plasma Membrane | other |
| **165** | NM_013328.2 | PYCR2 | pyrroline-5-carboxylate reductase family, member 2 | Cytoplasm | enzyme |
| **166** | NM_017659.3 | QPCTL | glutaminyl-peptide cyclotransferase-like | Cytoplasm | enzyme |
| **167** | NM_013277.3 | RACGAP1 | Rac GTPase activating protein 1 | Cytoplasm | transporter |
| **168** | NM_006325.3 | RAN | RAN, member RAS oncogene family | Nucleus | enzyme |
| **169** | NM_017805.2 | RASIP1 | Ras interacting protein 1 | Cytoplasm | other |
| **170** | NM_025232.2 | REEP4 | receptor accessory protein 4 | Other | other |
| **171** | NM_004296.4 | RGS6 | regulator of G-protein signaling 6 | Cytoplasm | enzyme |
| **172** | NM_014372.4 | RNF11 | ring finger protein 11 | Nucleus | other |
| **173** | NM_033251.1 | RPL13 | ribosomal protein L13 | Cytoplasm | other |
| **174** | NM_005698.2 | SCAMP3 | secretory carrier membrane protein 3 | Cytoplasm | transporter |
| **175** | NM_015670.4 | SENP3 | SUMO1/sentrin/SMT3 specific peptidase 3 | Nucleus | peptidase |
| **176** | NR_024271.1 | SEPT7P2 | septin 7 pseudogene 2 | Other | other |
| **177** | NM_006811.2 | SERINC3 | serine incorporator 3 | Cytoplasm | transporter |
| **178** | NM_144665.2 | SESN3 | sestrin 3 | Extracellular Space | other |
| **179** | NM_138484.2 | SGOL1 | shugoshin-like 1 (S. pombe) | Nucleus | other |
| **180** | NM_001042683.2 | SHPRH | SNF2 histone linker PHD RING helicase, E3 ubiquitin protein ligase | Nucleus | transcription regulator |
| **181** | NM_001040455.1 | SIDT2 | SID1 transmembrane family, member 2 | Cytoplasm | other |
| **182** | NM_173635.1 | SIGLECL1 | SIGLEC family like 1 | Other | other |
| **183** | NM_001029998.3 | SLC10A7 | solute carrier family 10 (sodium/bile acid cotransporter family), member 7 | Other | transporter |
| **184** | NM_000441.1 | SLC26A4 | solute carrier family 26, member 4 | Plasma Membrane | transporter |
| **185** | NM_144564.4 | SLC39A3 | solute carrier family 39 (zinc transporter), member 3 | Plasma Membrane | transporter |
| **186** | NM_003982.3 | SLC7A7 | solute carrier family 7 (amino acid transporter light chain, y+L system), member 7 | Plasma Membrane | transporter |
| **187** | NM_005445.3 | SMC3 | structural maintenance of chromosomes 3 | Nucleus | other |
| **188** | NM_006685.3 | SMR3B | submaxillary gland androgen regulated protein 3B | Nucleus | other |
| **189** | NM_003102.2 | SOD3 | superoxide dismutase 3, extracellular | Extracellular Space | enzyme |
| **190** | NM_017425.3 | SPA17 | sperm autoantigenic protein 17 | Plasma Membrane | other |
| **191** | NM_022827.2 | SPATA20 | spermatogenesis associated 20 | Other | other |
| **192** | NM_173499.3 | SPATA8 | spermatogenesis associated 8 | Other | other |
| **193** | NM_145658.3 | SPESP1 | sperm equatorial segment protein 1 | Cytoplasm | other |
| **194** | NM_003119.2 | SPG7 | spastic paraplegia 7 (pure and complicated autosomal recessive) | Cytoplasm | peptidase |
| **195** | NR_003062.1 | SPRR2C | small proline-rich protein 2C (pseudogene) | Cytoplasm | other |
| **196** | NM_021199.2 | SQRDL | sulfide quinone reductase-like (yeast) | Cytoplasm | enzyme |
| **197** | NM_012108.2 | STAP1 | signal transducing adaptor family member 1 | Cytoplasm | other |
| **198** | NM_173575.2 | STK32C | serine/threonine kinase 32C | Other | kinase |
| **199** | NM_013403.2 | STRN4 | striatin, calmodulin binding protein 4 | Cytoplasm | other |
| **200** | NM_003164.3 | STX5 | syntaxin 5 | Cytoplasm | transporter |
| **201** | NM_003849.3 | SUCLG1 | succinate-CoA ligase, alpha subunit | Cytoplasm | enzyme |
| **202** | NM_172231.2 | SUGP1 | SURP and G patch domain containing 1 | Nucleus | other |
| **203** | NM_053278.1 | TAAR8 | trace amine associated receptor 8 | Plasma Membrane | G-protein coupled receptor |
| **204** | NM_001008272.1 | TAGLN3 | transgelin 3 | Extracellular Space | other |
| **205** | NM_001082537.2 | TCTN1 | tectonic family member 1 | Extracellular Space | other |
| **206** | NM_198795.1 | TDRD1 | tudor domain containing 1 | Cytoplasm | other |
| **207** | NM_172000.3 | TEDDM1 | transmembrane epididymal protein 1 | Other | other |
| **208** | NM_003920.3 | TIMELESS | timeless circadian clock | Nucleus | other |
| **209** | NR_028383.1 | TIMM8B | translocase of inner mitochondrial membrane 8 homolog B (yeast) | Cytoplasm | transporter |
| **210** | NM_006405.5 | TM9SF1 | transmembrane 9 superfamily member 1 | Plasma Membrane | transporter |
| **211** | NM_022152.4 | TMBIM1 | transmembrane BAX inhibitor motif containing 1 | Cytoplasm | other |
| **212** | NM_016056.2 | TMBIM4 | transmembrane BAX inhibitor motif containing 4 | Nucleus | other |
| **213** | NM_194280.3 | TMEM219 | transmembrane protein 219 | Other | other |
| **214** | NM_182526.2 | TMEM229B | transmembrane protein 229B | Other | other |
| **215** | NM_018126.2 | TMEM33 | transmembrane protein 33 | Cytoplasm | other |
| **216** | NM_018112.1 | TMEM38B | transmembrane protein 38B | Nucleus | ion channel |
| **217** | NM_021156.2 | TMX4 | thioredoxin-related transmembrane protein 4 | Cytoplasm | enzyme |
| **218** | NM_003284.3 | TNP1 | transition protein 1 (during histone to protamine replacement) | Nucleus | other |
| **219** | NM_005486.2 | TOM1L1 | target of myb1 (chicken)-like 1 | Cytoplasm | other |
| **220** | NM_007027.3 | TOPBP1 | topoisomerase (DNA) II binding protein 1 | Nucleus | other |
| **221** | NM_025204.2 | TRABD | TraB domain containing | Extracellular Space | other |
| **222** | NM_014831.2 | TRANK1 | tetratricopeptide repeat and ankyrin repeat containing 1 | Nucleus | transcription regulator |
| **223** | NM_004237.3 | TRIP13 | thyroid hormone receptor interactor 13 | Cytoplasm | transcription regulator |
| **224** | NM_004600.5 | TROVE2 | TROVE domain family, member 2 | Nucleus | other |
| **225** | NM_015638.2 | TRPC4AP | transient receptor potential cation channel, subfamily C, member 4 associated protein | Cytoplasm | transporter |
| **226** | NM_001001410.2 | TSR3 | TSR3, 20S rRNA accumulation, homolog (S. cerevisiae) | Other | other |
| **227** | NM_015140.3 | TTLL12 | tubulin tyrosine ligase-like family, member 12 | Other | other |
| **228** | NM_207309.2 | UAP1L1 | UDP-N-acteylglucosamine pyrophosphorylase 1-like 1 | Other | other |
| **229** | NM_003969.3 | UBE2M | ubiquitin-conjugating enzyme E2M | Cytoplasm | enzyme |
| **230** | NM_014176.3 | UBE2T | ubiquitin-conjugating enzyme E2T (putative) | Nucleus | enzyme |
| **231** | NM_020765.2 | UBR4 | ubiquitin protein ligase E3 component n-recognin 4 | Nucleus | other |
| **232** | NM_003715.2 | USO1 | USO1 vesicle transport factor | Cytoplasm | transporter |
| **233** | NM_005151.3 | USP14 | ubiquitin specific peptidase 14 (tRNA-guanine transglycosylase) | Cytoplasm | peptidase |
| **234** | NM_004385.4 | VCAN | versican | Extracellular Space | other |
| **235** | NM_003375.3 | VDAC2 | voltage-dependent anion channel 2 | Cytoplasm | ion channel |
| **236** | NM_003381.2 | VIP | vasoactive intestinal peptide | Extracellular Space | other |
| **237** | NM_016312.2 | WBP11 | WW domain binding protein 11 | Nucleus | phosphatase |
| **238** | NM_080753.2 | WFDC10A | WAP four-disulfide core domain 10A | Extracellular Space | other |
| **239** | NM_033276.2 | XRCC6BP1 | XRCC6 binding protein 1 | Other | kinase |
| **240** | NM_001039617.1 | ZDHHC19 | zinc finger, DHHC-type containing 19 | Other | other |
| **241** | NM_053023.3 | ZFP91 | ZFP91 zinc finger protein | Nucleus | transcription regulator |
| **242** | NM_152338.2 | ZG16 | zymogen granule protein 16 | Extracellular Space | other |
| **243** | NM_018555.5 | ZNF331 | zinc finger protein 331 | Nucleus | other |
| **244** | NM_001101419.1 | ZNF541 | zinc finger protein 541 | Nucleus | other |
| **245** | NM_024303.1 | ZSCAN5A | zinc finger and SCAN domain containing 5A | Nucleus | transcription regulator |
| **246** | XR_038906.2 | unknown | unknown | unknown | unknown |
| **247** | XR_078588.1 | unknown | unknown | unknown | unknown |
