# Supplementary material for: Deregulation of sertoli and leydig cells function in patients with klinefelter syndrome as evidenced by testis transcriptome analysis
Source: BMC Genomics. 2015 Mar 7;16(1):156. doi: 10.1186/s12864-015-1356-0 (PMC4362638; doi:10.1186/s12864-015-1356-0)
Supplement: Additional file 3: Table S2. — Alphabetical list of the 656 up-expressed genes in Cluster B. [file 12864_2015_1356_MOESM3_ESM.docx]

Table S2: alphabetical list of the 656 up-expressed genes in Cluster B

|  | **ID** | **SYMBOL** | **ENTREZ GENE NAME** | **LOCATION** | **TYPE(S)** |
| --- | --- | --- | --- | --- | --- |
| **1** | NM_001122674.1 | ABCD3 | ATP-binding cassette, sub-family D (ALD), member 3 | Cytoplasm | transporter |
| **2** | NM_016006.4 | ABHD5 | abhydrolase domain containing 5 | Cytoplasm | enzyme |
| **3** | NM_015429.3 | ABI3BP | ABI family, member 3 (NESH) binding protein | Extracellular Space | other |
| **4** | NM_001003408.1 | ABLIM1 | actin binding LIM protein 1 | Cytoplasm | other |
| **5** | NM_014716.3 | ACAP1 | ArfGAP with coiled-coil, ankyrin repeat and PH domains 1 | Plasma Membrane | other |
| **6** | NM_001098.2 | ACO2 | aconitase 2, mitochondrial | Cytoplasm | enzyme |
| **7** | NM_025149.4 | ACSF2 | acyl-CoA synthetase family member 2 | Cytoplasm | enzyme |
| **8** | NM_004457.3 | ACSL3 | acyl-CoA synthetase long-chain family member 3 | Cytoplasm | enzyme |
| **9** | NM_003474.4 | ADAM12 | ADAM metallopeptidase domain 12 | Plasma Membrane | peptidase |
| **10** | NR_026938.2 | ADCY10P1 | adenylate cyclase 10 (soluble) pseudogene 1 | Other | other |
| **11** | NM_016282.2 | AK3 | adenylate kinase 3 | Cytoplasm | kinase |
| **12** | NM_004842.2 | AKAP7 | A kinase (PRKA) anchor protein 7 | Plasma Membrane | other |
| **13** | NM_001080538.2 | AKR1B15 | aldo-keto reductase family 1, member B15 | Other | other |
| **14** | NM_003739.4 | AKR1C3 | aldo-keto reductase family 1, member C3 | Cytoplasm | enzyme |
| **15** | NM_198867.1 | ALKBH6 | alkB, alkylation repair homolog 6 (E. coli) | Other | other |
| **16** | NM_023039.3 | ANKRA2 | ankyrin repeat, family A (RFXANK-like), 2 | Cytoplasm | transcription regulator |
| **17** | NM_052855.3 | ANKRD40 | ankyrin repeat domain 40 | Other | other |
| **18** | NM_000700.1 | ANXA1 | annexin A1 | Plasma Membrane | other |
| **19** | NM_001154.3 | ANXA5 | annexin A5 | Plasma Membrane | other |
| **20** | NM_001159.3 | AOX1 | aldehyde oxidase 1 | Cytoplasm | enzyme |
| **21** | NM_001127510.1 | APC | adenomatous polyposis coli | Nucleus | enzyme |
| **22** | NM_016022.2 | APH1A | APH1A gamma secretase subunit | Cytoplasm | peptidase |
| **23** | NM_015957.2 | APIP | APAF1 interacting protein | Cytoplasm | enzyme |
| **24** | NM_000042.2 | APOH | apolipoprotein H (beta-2-glycoprotein I) | Extracellular Space | transporter |
| **25** | NM_018011.3 | ARGLU1 | arginine and glutamate rich 1 | Other | other |
| **26** | NM_024605.3 | ARHGAP10 | Rho GTPase activating protein 10 | Cytoplasm | other |
| **27** | NM_020754.2 | ARHGAP31 | Rho GTPase activating protein 31 | Cytoplasm | other |
| **28** | NM_032496.2 | ARHGAP9 | Rho GTPase activating protein 9 | Cytoplasm | other |
| **29** | NM_017519.2 | ARID1B | AT rich interactive domain 1B (SWI1-like) | Nucleus | transcription regulator |
| **30** | NM_006407.3 | ARL6IP5 | ADP-ribosylation-like factor 6 interacting protein 5 | Cytoplasm | other |
| **31** | NM_138795.2 | ARL8A | ADP-ribosylation factor-like 8A | Cytoplasm | enzyme |
| **32** | NM_183376.2 | ARRDC4 | arrestin domain containing 4 | Other | other |
| **33** | NM_001127505.1 | ASAH1 | N-acylsphingosine amidohydrolase (acid ceramidase) 1 | Cytoplasm | enzyme |
| **34** | NM_130387.5 | ASB14 | ankyrin repeat and SOCS box containing 14 | Other | other |
| **35** | NM_018489.2 | ASH1L | ash1 (absent, small, or homeotic)-like (Drosophila) | Nucleus | transcription regulator |
| **36** | NM_052936.2 | ATG4A | autophagy related 4A, cysteine peptidase | Cytoplasm | peptidase |
| **37** | NM_005174.2 | ATP5C1 | ATP synthase, H+ transporting, mitochondrial F1 complex, gamma polypeptide 1 | Cytoplasm | transporter |
| **38** | NM_001694.2 | ATP6V0C | ATPase, H+ transporting, lysosomal 16kDa, V0 subunit c | Cytoplasm | transporter |
| **39** | NM_181575.3 | AUP1 | ancient ubiquitous protein 1 | Cytoplasm | other |
| **40** | NM_004048.2 | B2M | beta-2-microglobulin | Plasma Membrane | transmembrane receptor |
| **41** | NM_018644.3 | B3GAT1 | beta-1,3-glucuronyltransferase 1 (glucuronosyltransferase P) | Cytoplasm | enzyme |
| **42** | NM_031885.3 | BBS2 | Bardet-Biedl syndrome 2 | Cytoplasm | other |
| **43** | NM_001170715.1 | BCAR1 | breast cancer anti-estrogen resistance 1 | Plasma Membrane | enzyme |
| **44** | NM_004183.3 | BEST1 | bestrophin 1 | Plasma Membrane | ion channel |
| **45** | NM_004052.2 | BNIP3 | BCL2/adenovirus E1B 19kDa interacting protein 3 | Cytoplasm | other |
| **46** | NM_004331.2 | BNIP3L | BCL2/adenovirus E1B 19kDa interacting protein 3-like | Cytoplasm | other |
| **47** | NM_148894.2 | BOD1L1 | biorientation of chromosomes in cell division 1-like 1 | Extracellular Space | other |
| **48** | NR_026649.1 | BPHL | biphenyl hydrolase-like (serine hydrolase) | Cytoplasm | enzyme |
| **49** | NM_007371.3 | BRD3 | bromodomain containing 3 | Nucleus | kinase |
| **50** | NM_001009877.2 | BRD9 | bromodomain containing 9 | Other | other |
| **51** | NM_181443.1 | BTBD3 | BTB (POZ) domain containing 3 | Other | other |
| **52** | NM_006763.2 | BTG2 | BTG family, member 2 | Nucleus | transcription regulator |
| **53** | NM_020643.2 | C11orf16 | chromosome 11 open reading frame 16 | Other | other |
| **54** | NM_080659.2 | C11orf52 | chromosome 11 open reading frame 52 | Other | other |
| **55** | NM_001080406.1 | C12orf71 | chromosome 12 open reading frame 71 | Other | other |
| **56** | NM_001130706.1 | C14orf93 | chromosome 14 open reading frame 93 | Other | other |
| **57** | NM_052849.2 | C15orf57 | chromosome 15 open reading frame 57 | Other | other |
| **58** | NR_027379.1 | C1orf180 | chromosome 1 open reading frame 180 | Other | other |
| **59** | NM_001085375.1 | C1orf226 | chromosome 1 open reading frame 226 | Other | other |
| **60** | NM_024579.3 | C1orf54 | chromosome 1 open reading frame 54 | Other | other |
| **61** | NM_001733.4 | C1R | complement component 1, r subcomponent | Extracellular Space | peptidase |
| **62** | NM_080616.3 | C20orf112 | chromosome 20 open reading frame 112 | Other | other |
| **63** | NM_001105519.1 | C2orf70 | chromosome 2 open reading frame 70 | Other | other |
| **64** | NM_001735.2 | C5 | complement component 5 | Extracellular Space | cytokine |
| **65** | NM_175921.4 | C5orf51 | chromosome 5 open reading frame 51 | Other | other |
| **66** | NM_203459.1 | CAMSAP2 | calmodulin regulated spectrin-associated protein family, member 2 | Other | other |
| **67** | NM_006136.2 | CAPZA2 | capping protein (actin filament) muscle Z-line, alpha 2 | Cytoplasm | other |
| **68** | NM_001172895.1 | CAV1 | caveolin 1, caveolae protein, 22kDa | Plasma Membrane | transmembrane receptor |
| **69** | NM_001233.3 | CAV2 | caveolin 2 | Plasma Membrane | other |
| **70** | NM_145020.3 | CCDC11 | coiled-coil domain containing 11 | Other | other |
| **71** | NM_144719.3 | CCDC13 | coiled-coil domain containing 13 | Other | other |
| **72** | NM_001144872.1 | CCDC42B | coiled-coil domain containing 42B | Other | other |
| **73** | NM_004060.3 | CCNG1 | cyclin G1 | Nucleus | other |
| **74** | NR_015382.1 | CD27-AS1 | CD27 antisense RNA 1 | Other | other |
| **75** | NM_006725.3 | CD6 | CD6 molecule | Plasma Membrane | transmembrane receptor |
| **76** | NM_001040034.1 | CD63 | CD63 molecule | Plasma Membrane | other |
| **77** | NM_033543.3 | CEACAM21 | carcinoembryonic antigen-related cell adhesion molecule 21 | Other | other |
| **78** | NM_006890.3 | CEACAM7 | carcinoembryonic antigen-related cell adhesion molecule 7 | Plasma Membrane | other |
| **79** | NM_005194.2 | CEBPB | CCAAT/enhancer binding protein (C/EBP), beta | Nucleus | transcription regulator |
| **80** | NM_005195.3 | CEBPD | CCAAT/enhancer binding protein (C/EBP), delta | Nucleus | transcription regulator |
| **81** | NM_001012507.2 | CENPW | centromere protein W | Nucleus | other |
| **82** | NM_025114.3 | CEP290 | centrosomal protein 290kDa | Cytoplasm | transcription regulator |
| **83** | NM_004344.1 | CETN2 | centrin, EF-hand protein, 2 | Nucleus | enzyme |
| **84** | NM_033378.1 | CGB2 | chorionic gonadotropin, beta polypeptide 2 | Other | other |
| **85** | NM_001821.3 | CHML | choroideremia-like (Rab escort protein 2) | Cytoplasm | enzyme |
| **86** | NM_015892.2 | CHST15 | carbohydrate (N-acetylgalactosamine 4-sulfate 6-O) sulfotransferase 15 | Plasma Membrane | enzyme |
| **87** | NM_014918.4 | CHSY1 | chondroitin sulfate synthase 1 | Cytoplasm | enzyme |
| **88** | NM_145165.2 | CHURC1 | churchill domain containing 1 | Nucleus | transcription regulator |
| **89** | NR_023313.1 | CIRBP | cold inducible RNA binding protein | Nucleus | translation regulator |
| **90** | NM_006079.3 | CITED2 | Cbp/p300-interacting transactivator, with Glu/Asp-rich carboxy-terminal domain, 2 | Nucleus | transcription regulator |
| **91** | NM_000084.2 | CLCN5 | chloride channel, voltage-sensitive 5 | Plasma Membrane | ion channel |
| **92** | NM_006580.3 | CLDN16 | claudin 16 | Plasma Membrane | transporter |
| **93** | NM_003277.3 | CLDN5 | claudin 5 | Plasma Membrane | other |
| **94** | NM_001042432.1 | CLN3 | ceroid-lipofuscinosis, neuronal 3 | Cytoplasm | other |
| **95** | NM_006660.3 | CLPX | ClpX caseinolytic peptidase X homolog (E. coli) | Cytoplasm | enzyme |
| **96** | NM_015386.2 | COG4 | component of oligomeric golgi complex 4 | Cytoplasm | transporter |
| **97** | NM_020882.2 | COL20A1 | collagen, type XX, alpha 1 | Extracellular Space | other |
| **98** | NM_000093.3 | COL5A1 | collagen, type V, alpha 1 | Extracellular Space | other |
| **99** | NM_057165.4 | COL6A3 | collagen, type VI, alpha 3 | Extracellular Space | other |
| **100** | NM_001850.3 | COL8A1 | collagen, type VIII, alpha 1 | Extracellular Space | other |
| **101** | NM_017421.3 | COQ3 | coenzyme Q3 methyltransferase | Cytoplasm | enzyme |
| **102** | NM_004074.2 | COX8A | cytochrome c oxidase subunit VIIIA (ubiquitous) | Cytoplasm | enzyme |
| **103** | NM_003909.3 | CPNE3 | copine III | Cytoplasm | kinase |
| **104** | NM_016134.2 | CPQ | carboxypeptidase Q | Extracellular Space | peptidase |
| **105** | NM_006693.2 | CPSF4 | cleavage and polyadenylation specific factor 4, 30kDa | Nucleus | other |
| **106** | NM_182898.2 | CREB5 | cAMP responsive element binding protein 5 | Nucleus | transcription regulator |
| **107** | NM_006891.3 | CRYGD | crystallin, gamma D | Cytoplasm | other |
| **108** | NM_033225.5 | CSMD1 | CUB and Sushi multiple domains 1 | Plasma Membrane | other |
| **109** | NM_001893.3 | CSNK1D | casein kinase 1, delta | Cytoplasm | kinase |
| **110** | NM_006574.3 | CSPG5 | chondroitin sulfate proteoglycan 5 (neuroglycan C) | Extracellular Space | growth factor |
| **111** | NM_000100.2 | CSTB | cystatin B (stefin B) | Cytoplasm | peptidase |
| **112** | NM_001328.2 | CTBP1 | C-terminal binding protein 1 | Nucleus | enzyme |
| **113** | NM_138455.2 | CTHRC1 | collagen triple helix repeat containing 1 | Extracellular Space | other |
| **114** | NM_001903.2 | CTNNA1 | catenin (cadherin-associated protein), alpha 1, 102kDa | Plasma Membrane | other |
| **115** | NM_145918.2 | CTSL | cathepsin L | Cytoplasm | peptidase |
| **116** | NM_000634.2 | CXCR1 | chemokine (C-X-C motif) receptor 1 | Plasma Membrane | G-protein coupled receptor |
| **117** | NM_178124.4 | CXorf40A/CXorf40B | chromosome X open reading frame 40A | Other | other |
| **118** | NM_148923.2 | CYB5A | cytochrome b5 type A (microsomal) | Cytoplasm | enzyme |
| **119** | NM_001914.2 | CYB5A | cytochrome b5 type A (microsomal) | Cytoplasm | enzyme |
| **120** | NM_030579.2 | CYB5B | cytochrome b5 type B (outer mitochondrial membrane) | Cytoplasm | enzyme |
| **121** | NM_134268.3 | CYGB | cytoglobin | Cytoplasm | transporter |
| **122** | NR_001278.1 | CYP2B7P1 | cytochrome P450, family 2, subfamily B, polypeptide 7 pseudogene 1 | Other | enzyme |
| **123** | NM_001349.2 | DARS | aspartyl-tRNA synthetase | Cytoplasm | enzyme |
| **124** | NM_018122.4 | DARS2 | aspartyl-tRNA synthetase 2, mitochondrial | Cytoplasm | enzyme |
| **125** | NM_001029865.1 | DBX1 | developing brain homeobox 1 | Nucleus | transcription regulator |
| **126** | NM_133507.2 | DCN | decorin | Extracellular Space | other |
| **127** | NM_023935.1 | DDRGK1 | DDRGK domain containing 1 | Extracellular Space | other |
| **128** | NM_004939.1 | DDX1 | DEAD (Asp-Glu-Ala-Asp) box helicase 1 | Nucleus | enzyme |
| **129** | NM_001012967.1 | DDX60L | DEAD (Asp-Glu-Ala-Asp) box polypeptide 60-like | Other | other |
| **130** | NM_001040705.1 | DEFB107A/DEFB107B | defensin, beta 107A | Extracellular Space | other |
| **131** | NM_003677.3 | DENR | density-regulated protein | Other | other |
| **132** | NR_026645.1 | DET1 | de-etiolated homolog 1 (Arabidopsis) | Nucleus | other |
| **133** | NM_033081.1 | DIDO1 | death inducer-obliterator 1 | Nucleus | other |
| **134** | NM_001362.3 | DIO3 | deiodinase, iodothyronine, type III | Plasma Membrane | enzyme |
| **135** | NM_000108.3 | DLD | dihydrolipoamide dehydrogenase | Cytoplasm | enzyme |
| **136** | NM_005509.4 | DMXL1 | Dmx-like 1 | Extracellular Space | other |
| **137** | NM_001002762.2 | DNAJB12 | DnaJ (Hsp40) homolog, subfamily B, member 12 | Cytoplasm | other |
| **138** | NM_001031723.2 | DNAJB14 | DnaJ (Hsp40) homolog, subfamily B, member 14 | Other | enzyme |
| **139** | NM_013238.2 | DNAJC15 | DnaJ (Hsp40) homolog, subfamily C, member 15 | Cytoplasm | other |
| **140** | NM_018163.2 | DNAJC17 | DnaJ (Hsp40) homolog, subfamily C, member 17 | Other | other |
| **141** | NM_004944.2 | DNASE1L3 | deoxyribonuclease I-like 3 | Nucleus | enzyme |
| **142** | NM_001005336.1 | DNM1 | dynamin 1 | Cytoplasm | enzyme |
| **143** | NM_000110.3 | DPYD | dihydropyrimidine dehydrogenase | Cytoplasm | enzyme |
| **144** | NM_006870.3 | DSTN | destrin (actin depolymerizing factor) | Cytoplasm | other |
| **145** | NM_004417.3 | DUSP1 | dual specificity phosphatase 1 | Nucleus | phosphatase |
| **146** | NM_030640.2 | DUSP16 | dual specificity phosphatase 16 | Nucleus | phosphatase |
| **147** | NM_001080463.1 | DYNC2H1 | dynein, cytoplasmic 2, heavy chain 1 | Cytoplasm | other |
| **148** | NM_014183.2 | DYNLRB1 | dynein, light chain, roadblock-type 1 | Cytoplasm | other |
| **149** | NM_001004023.1 | DYRK3 | dual-specificity tyrosine-(Y)-phosphorylation regulated kinase 3 | Nucleus | kinase |
| **150** | NM_005755.2 | EBI3 | Epstein-Barr virus induced 3 | Extracellular Space | cytokine |
| **151** | NM_007265.2 | ECD | ecdysoneless homolog (Drosophila) | Nucleus | transcription regulator |
| **152** | NM_013302.3 | EEF2K | eukaryotic elongation factor-2 kinase | Cytoplasm | kinase |
| **153** | NM_014335.2 | EID1 | EP300 interacting inhibitor of differentiation 1 | Nucleus | transcription regulator |
| **154** | NM_004836.5 | EIF2AK3 | eukaryotic translation initiation factor 2-alpha kinase 3 | Cytoplasm | kinase |
| **155** | NM_001415.3 | EIF2S3 | eukaryotic translation initiation factor 2, subunit 3 gamma, 52kDa | Cytoplasm | translation regulator |
| **156** | NM_001431.3 | EPB41L2 | erythrocyte membrane protein band 4.1-like 2 | Plasma Membrane | other |
| **157** | NM_015630.3 | EPC2 | enhancer of polycomb homolog 2 (Drosophila) | Other | other |
| **158** | NR_027948.1 | ERC1 | ELKS/RAB6-interacting/CAST family member 1 | Cytoplasm | other |
| **159** | NM_001162422.1 | ETS1 | v-ets erythroblastosis virus E26 oncogene homolog 1 (avian) | Nucleus | transcription regulator |
| **160** | NM_014612.3 | FAM120A | family with sequence similarity 120A | Cytoplasm | other |
| **161** | NM_017848.4 | FAM120C | family with sequence similarity 120C | Other | other |
| **162** | NM_001008226.1 | FAM154B | family with sequence similarity 154, member B | Other | other |
| **163** | NM_001009993.2 | FAM168B | family with sequence similarity 168, member B | Other | other |
| **164** | NM_023933.1 | FAM173A | family with sequence similarity 173, member A | Other | other |
| **165** | NM_018121.3 | FAM178A | family with sequence similarity 178, member A | Other | other |
| **166** | NM_173563.1 | FAM217A | family with sequence similarity 217, member A | Extracellular Space | other |
| **167** | NM_001083537.1 | FAM86B1 | family with sequence similarity 86, member B1 | Other | other |
| **168** | NM_016255.2 | FAM8A1 | family with sequence similarity 8, member A1 | Other | other |
| **169** | NM_001136572.1 | FAM90A10P/FAM90A7P | putative protein FAM90A7 | Other | other |
| **170** | NM_032385.3 | FAXDC2 | fatty acid hydroxylase domain containing 2 | Other | other |
| **171** | NM_032447.3 | FBN3 | fibrillin 3 | Extracellular Space | other |
| **172** | NM_001029860.2 | FBXO43 | F-box protein 43 | Nucleus | other |
| **173** | NM_004109.3 | FDX1 | ferredoxin 1 | Cytoplasm | transporter |
| **174** | NM_006832.2 | FERMT2 | fermitin family member 2 | Cytoplasm | other |
| **175** | NM_021939.3 | FKBP10 | FK506 binding protein 10, 65 kDa | Cytoplasm | enzyme |
| **176** | NM_002013.3 | FKBP3 | FK506 binding protein 3, 25kDa | Nucleus | enzyme |
| **177** | NR_027339.1 | FKBP9L | FK506 binding protein 9-like | Other | other |
| **178** | XR_078551.1 | FLJ30403 | uncharacterized LOC729975 | Other | other |
| **179** | NR_015360.1 | FLJ33630 | uncharacterized LOC644873 | Other | other |
| **180** | NM_001453.2 | FOXC1 | forkhead box C1 | Nucleus | transcription regulator |
| **181** | NM_003923.2 | FOXH1 | forkhead box H1 | Nucleus | transcription regulator |
| **182** | NM_181721.2 | FOXR1 | forkhead box R1 | Nucleus | transcription regulator |
| **183** | NM_031904.3 | FRMD8 | FERM domain containing 8 | Other | other |
| **184** | NR_002201.1 | FTH1P3 | ferritin, heavy polypeptide 1 pseudogene 3 | Other | other |
| **185** | NM_000146.3 | FTL | ferritin, light polypeptide | Cytoplasm | enzyme |
| **186** | NR_028388.2 | FUS | fused in sarcoma | Nucleus | transcription regulator |
| **187** | NM_021603.3 | FXYD2 | FXYD domain containing ion transport regulator 2 | Plasma Membrane | ion channel |
| **188** | NM_002040.3 | GABPA | GA binding protein transcription factor, alpha subunit 60kDa | Nucleus | transcription regulator |
| **189** | NM_001037525.1 | GALC | galactosylceramidase | Cytoplasm | enzyme |
| **190** | NR_023348.1 | GAS8 | growth arrest-specific 8 | Cytoplasm | other |
| **191** | NM_004293.3 | GDA | guanine deaminase | Cytoplasm | enzyme |
| **192** | NM_001496.3 | GFRA3 | GDNF family receptor alpha 3 | Plasma Membrane | transmembrane receptor |
| **193** | NM_014394.2 | GHITM | growth hormone inducible transmembrane protein | Cytoplasm | other |
| **194** | NM_018384.3 | GIMAP5 | GTPase, IMAP family member 5 | Cytoplasm | other |
| **195** | NM_057170.3 | GIT2 | G protein-coupled receptor kinase interacting ArfGAP 2 | Nucleus | other |
| **196** | NM_015554.1 | GLCE | glucuronic acid epimerase | Cytoplasm | enzyme |
| **197** | NM_022343.2 | GLIPR2 | GLI pathogenesis-related 2 | Cytoplasm | other |
| **198** | NM_004246.1 | GLP2R | glucagon-like peptide 2 receptor | Plasma Membrane | G-protein coupled receptor |
| **199** | NM_016433.3 | GLTP | glycolipid transfer protein | Cytoplasm | transporter |
| **200** | NM_004877.2 | GMFG | glia maturation factor, gamma | Cytoplasm | growth factor |
| **201** | NM_021629.3 | GNB4 | guanine nucleotide binding protein (G protein), beta polypeptide 4 | Plasma Membrane | enzyme |
| **202** | NM_016194.3 | GNB5 | guanine nucleotide binding protein (G protein), beta 5 | Plasma Membrane | enzyme |
| **203** | NM_001184819.1 | GNL3L | guanine nucleotide binding protein-like 3 (nucleolar)-like | Nucleus | other |
| **204** | NM_018066.3 | GPN2 | GPN-loop GTPase 2 | Other | other |
| **205** | NM_025048.2 | GPR110 | G protein-coupled receptor 110 | Plasma Membrane | G-protein coupled receptor |
| **206** | NM_005290.1 | GPR15 | G protein-coupled receptor 15 | Plasma Membrane | G-protein coupled receptor |
| **207** | NM_005299.2 | GPR31 | G protein-coupled receptor 31 | Plasma Membrane | G-protein coupled receptor |
| **208** | NM_022036.2 | GPRC5C | G protein-coupled receptor, family C, group 5, member C | Plasma Membrane | G-protein coupled receptor |
| **209** | NM_198182.2 | GRHL1 | grainyhead-like 1 (Drosophila) | Nucleus | transcription regulator |
| **210** | NM_012203.1 | GRHPR | glyoxylate reductase/hydroxypyruvate reductase | Cytoplasm | enzyme |
| **211** | NM_175611.2 | GRIK1 | glutamate receptor, ionotropic, kainate 1 | Plasma Membrane | ion channel |
| **212** | NM_001127662.1 | GSN | gelsolin | Extracellular Space | other |
| **213** | NM_000846.4 | GSTA2 | glutathione S-transferase alpha 2 | Cytoplasm | enzyme |
| **214** | NM_000846.4 | GSTA2 | glutathione S-transferase alpha 2 | Cytoplasm | enzyme |
| **215** | NM_000852.3 | GSTP1 | glutathione S-transferase pi 1 | Cytoplasm | enzyme |
| **216** | NM_002101.3 | GYPC | glycophorin C (Gerbich blood group) | Plasma Membrane | other |
| **217** | NM_002106.3 | H2AFZ | H2A histone family, member Z | Nucleus | other |
| **218** | NM_032124.4 | HDHD2 | haloacid dehalogenase-like hydrolase domain containing 2 | Other | other |
| **219** | NR_002824.3 | HERC2P2 | hect domain and RLD 2 pseudogene 2 | Other | other |
| **220** | NM_022475.1 | HHIP | hedgehog interacting protein | Plasma Membrane | other |
| **221** | NM_198047.2 | HIBCH | 3-hydroxyisobutyryl-CoA hydrolase | Cytoplasm | enzyme |
| **222** | NM_001098202.1 | HIC1 | hypermethylated in cancer 1 | Nucleus | transcription regulator |
| **223** | NM_021062.2 | HIST1H2BB | histone cluster 1, H2bb | Nucleus | other |
| **224** | NM_019111.3 | HLA-DRA | major histocompatibility complex, class II, DR alpha | Plasma Membrane | transmembrane receptor |
| **225** | NM_001098478.1 | HLA-F | major histocompatibility complex, class I, F | Plasma Membrane | transmembrane receptor |
| **226** | XM_001726942.2 | HMCN2 | hemicentin 2 | Extracellular Space | other |
| **227** | NM_001098272.1 | HMGCS1 | 3-hydroxy-3-methylglutaryl-CoA synthase 1 (soluble) | Cytoplasm | enzyme |
| **228** | NM_005517.3 | HMGN2 | high mobility group nucleosomal binding domain 2 | Nucleus | other |
| **229** | NM_031243.2 | HNRNPA2B1 | heterogeneous nuclear ribonucleoprotein A2/B1 | Nucleus | other |
| **230** | NM_031314.2 | HNRNPC | heterogeneous nuclear ribonucleoprotein C (C1/C2) | Nucleus | other |
| **231** | NM_031369.2 | HNRNPD | heterogeneous nuclear ribonucleoprotein D (AU-rich element RNA binding protein 1, 37kDa) | Nucleus | transcription regulator |
| **232** | NR_003249.1 | HNRNPDL | heterogeneous nuclear ribonucleoprotein D-like | Nucleus | other |
| **233** | NM_005520.2 | HNRNPH1 | heterogeneous nuclear ribonucleoprotein H1 (H) | Nucleus | other |
| **234** | NR_027297.1 | HOMER3 | homer homolog 3 (Drosophila) | Plasma Membrane | other |
| **235** | NM_013312.2 | HOOK2 | hook homolog 2 (Drosophila) | Cytoplasm | other |
| **236** | NM_014213.3 | HOXD9 | homeobox D9 | Nucleus | transcription regulator |
| **237** | NM_000413.2 | HSD17B1 | hydroxysteroid (17-beta) dehydrogenase 1 | Cytoplasm | enzyme |
| **238** | NM_000197.1 | HSD17B3 | hydroxysteroid (17-beta) dehydrogenase 3 | Cytoplasm | enzyme |
| **239** | NM_000414.2 | HSD17B4 | hydroxysteroid (17-beta) dehydrogenase 4 | Cytoplasm | enzyme |
| **240** | NM_003725.2 | HSD17B6 | hydroxysteroid (17-beta) dehydrogenase 6 | Other | enzyme |
| **241** | NM_016400.2 | HYPK | huntingtin interacting protein K | Cytoplasm | other |
| **242** | NM_012092.3 | ICOS | inducible T-cell co-stimulator | Plasma Membrane | transmembrane receptor |
| **243** | NM_015259.4 | ICOSLG | inducible T-cell co-stimulator ligand | Plasma Membrane | other |
| **244** | NM_004969.3 | IDE | insulin-degrading enzyme | Extracellular Space | peptidase |
| **245** | NM_004907.2 | IER2 | immediate early response 2 | Cytoplasm | other |
| **246** | NM_005533.3 | IFI35 | interferon-induced protein 35 | Nucleus | other |
| **247** | NM_022872.2 | IFI6 | interferon, alpha-inducible protein 6 | Cytoplasm | other |
| **248** | NM_003641.3 | IFITM1 | interferon induced transmembrane protein 1 | Plasma Membrane | transmembrane receptor |
| **249** | NM_002178.2 | IGFBP6 | insulin-like growth factor binding protein 6 | Extracellular Space | other |
| **250** | NM_153481.1 | IL17RE | interleukin 17 receptor E | Other | other |
| **251** | NM_014214.1 | IMPA2 | inositol(myo)-1(or 4)-monophosphatase 2 | Cytoplasm | phosphatase |
| **252** | NM_000884.2 | IMPDH2 | IMP (inosine 5'-monophosphate) dehydrogenase 2 | Cytoplasm | enzyme |
| **253** | NM_002192.2 | INHBA | inhibin, beta A | Extracellular Space | growth factor |
| **254** | NM_017759.4 | INO80D | INO80 complex subunit D | Other | other |
| **255** | NM_001017915.1 | INPP5D | inositol polyphosphate-5-phosphatase, 145kDa | Cytoplasm | phosphatase |
| **256** | NR_003512.1 | INS-IGF2 | INS-IGF2 readthrough | Other | other |
| **257** | NM_016048.2 | ISOC1 | isochorismatase domain containing 1 | Cytoplasm | enzyme |
| **258** | NM_002203.3 | ITGA2 | integrin, alpha 2 (CD49B, alpha 2 subunit of VLA-2 receptor) | Plasma Membrane | transmembrane receptor |
| **259** | NM_002213.3 | ITGB5 | integrin, beta 5 | Plasma Membrane | other |
| **260** | NM_033397.2 | ITPRIP | inositol 1,4,5-trisphosphate receptor interacting protein | Extracellular Space | other |
| **261** | NM_001034841.3 | ITPRIPL2 | inositol 1,4,5-trisphosphate receptor interacting protein-like 2 | Other | other |
| **262** | NM_152405.4 | JMY | junction mediating and regulatory protein, p53 cofactor | Nucleus | transcription regulator |
| **263** | NM_002228.3 | JUN | jun proto-oncogene | Nucleus | transcription regulator |
| **264** | NM_172375.1 | KCNH5 | potassium voltage-gated channel, subfamily H (eag-related), member 5 | Plasma Membrane | ion channel |
| **265** | NM_004983.2 | KCNJ9 | potassium inwardly-rectifying channel, subfamily J, member 9 | Plasma Membrane | ion channel |
| **266** | NM_001135914.1 | KCP | kielin/chordin-like protein | Extracellular Space | other |
| **267** | NM_138444.3 | KCTD12 | potassium channel tetramerization domain containing 12 | Plasma Membrane | ion channel |
| **268** | NM_001080424.1 | KDM6B | lysine (K)-specific demethylase 6B | Extracellular Space | other |
| **269** | NM_002035.2 | KDSR | 3-ketodihydrosphingosine reductase | Plasma Membrane | enzyme |
| **270** | NM_014846.3 | KIAA0196 | KIAA0196 | Cytoplasm | other |
| **271** | NM_018689.1 | KIAA1199 | KIAA1199 | Cytoplasm | other |
| **272** | NM_018330.5 | KIAA1598 | KIAA1598 | Other | other |
| **273** | NM_004521.2 | KIF5B | kinesin family member 5B | Cytoplasm | other |
| **274** | NM_032123.5 | KIRREL2 | kin of IRRE like 2 (Drosophila) | Plasma Membrane | other |
| **275** | NM_005552.4 | KLC1 | kinesin light chain 1 | Cytoplasm | other |
| **276** | NM_016270.2 | KLF2 | Kruppel-like factor 2 (lung) | Nucleus | transcription regulator |
| **277** | NM_181535.3 | KRT28 | keratin 28 | Other | other |
| **278** | NM_005554.3 | KRT6A | keratin 6A | Plasma Membrane | other |
| **279** | NM_175053.3 | KRT74 | keratin 74 | Other | other |
| **280** | NM_181621.3 | KRTAP13-2 | keratin associated protein 13-2 | Other | other |
| **281** | NM_016027.2 | LACTB2 | lactamase, beta 2 | Cytoplasm | other |
| **282** | NM_001127641.1 | LAMB3 | laminin, beta 3 | Extracellular Space | transporter |
| **283** | NM_006059.3 | LAMC3 | laminin, gamma 3 | Extracellular Space | other |
| **284** | NM_021970.3 | LAMTOR3 | late endosomal/lysosomal adaptor, MAPK and MTOR activator 3 | Cytoplasm | other |
| **285** | NM_000229.1 | LCAT | lecithin-cholesterol acyltransferase | Extracellular Space | enzyme |
| **286** | NM_178428.3 | LCE2A | late cornified envelope 2A | Other | other |
| **287** | NM_000527.3 | LDLR | low density lipoprotein receptor | Plasma Membrane | transporter |
| **288** | NM_005779.2 | LHFPL2 | lipoma HMGIC fusion partner-like 2 | Other | enzyme |
| **289** | NM_005569.3 | LIMK2 | LIM domain kinase 2 | Cytoplasm | kinase |
| **290** | NM_004987.3 | LIMS1 | LIM and senescent cell antigen-like domains 1 | Plasma Membrane | other |
| **291** | XR_078657.1 | LINC00460 | long intergenic non-protein coding RNA 460 | Other | other |
| **292** | NR_023925.1 | LINC00470 | long intergenic non-protein coding RNA 470 | Other | other |
| **293** | NR_023925.1 | LINC00470 | long intergenic non-protein coding RNA 470 | Other | other |
| **294** | NR_026796.1 | LINC00520 | long intergenic non-protein coding RNA 520 | Other | other |
| **295** | NM_001127605.1 | LIPA | lipase A, lysosomal acid, cholesterol esterase | Cytoplasm | enzyme |
| **296** | NR_024412.1 | LOC401397 | uncharacterized LOC401397 | Other | other |
| **297** | NM_001146181.1 | LOC649330 | heterogeneous nuclear ribonucleoprotein C-like | Other | other |
| **298** | NM_031490.2 | LONP2 | lon peptidase 2, peroxisomal | Cytoplasm | peptidase |
| **299** | NM_014873.2 | LPGAT1 | lysophosphatidylglycerol acyltransferase 1 | Cytoplasm | other |
| **300** | NM_001135772.1 | LRP5L | low density lipoprotein receptor-related protein 5-like | Other | other |
| **301** | NM_001099678.1 | LRRC58 | leucine rich repeat containing 58 | Other | other |
| **302** | NM_001145310.1 | LRTOMT | leucine rich transmembrane and 0-methyltransferase domain containing | Other | enzyme |
| **303** | NM_015578.2 | LSM14A | LSM14A, SCD6 homolog A (S. cerevisiae) | Cytoplasm | other |
| **304** | NM_001001438.2 | LSS | lanosterol synthase (2,3-oxidosqualene-lanosterol cyclase) | Cytoplasm | enzyme |
| **305** | NM_206943.2 | LTBP1 | latent transforming growth factor beta binding protein 1 | Extracellular Space | other |
| **306** | NM_016424.4 | LUC7L3 | LUC7-like 3 (S. cerevisiae) | Nucleus | other |
| **307** | NM_001033667.1 | LY9 | lymphocyte antigen 9 | Plasma Membrane | other |
| **308** | NM_177476.2 | LYNX1 | Ly6/neurotoxin 1 | Plasma Membrane | transporter |
| **309** | NM_007260.2 | LYPLA2 | lysophospholipase II | Cytoplasm | enzyme |
| **310** | NM_001136543.1 | LYSMD1 | LysM, putative peptidoglycan-binding, domain containing 1 | Other | other |
| **311** | NM_001005332.1 | MAGED1 | melanoma antigen family D, 1 | Plasma Membrane | transcription regulator |
| **312** | NM_001033057.1 | MAGI1 | membrane associated guanylate kinase, WW and PDZ domain containing 1 | Plasma Membrane | kinase |
| **313** | NM_002371.2 | MAL | mal, T-cell differentiation protein | Plasma Membrane | transporter |
| **314** | NR_002819.2 | MALAT1 | metastasis associated lung adenocarcinoma transcript 1 (non-protein coding) | Nucleus | other |
| **315** | NR_002819.2 | MALAT1 | metastasis associated lung adenocarcinoma transcript 1 (non-protein coding) | Nucleus | other |
| **316** | NM_001177466.1 | MAMLD1 | mastermind-like domain containing 1 | Other | other |
| **317** | NM_005907.2 | MAN1A1 | mannosidase, alpha, class 1A, member 1 | Cytoplasm | enzyme |
| **318** | NM_005909.3 | MAP1B | microtubule-associated protein 1B | Cytoplasm | other |
| **319** | NM_005909.3 | MAP1B | microtubule-associated protein 1B | Cytoplasm | other |
| **320** | NM_001039538.1 | MAP2 | microtubule-associated protein 2 | Plasma Membrane | other |
| **321** | NM_002758.3 | MAP2K6 | mitogen-activated protein kinase kinase 6 | Cytoplasm | kinase |
| **322** | NM_003954.2 | MAP3K14 | mitogen-activated protein kinase kinase kinase 14 | Cytoplasm | kinase |
| **323** | NM_004672.3 | MAP3K6 | mitogen-activated protein kinase kinase kinase 6 | Other | kinase |
| **324** | NM_004834.3 | MAP4K4 | mitogen-activated protein kinase kinase kinase kinase 4 | Cytoplasm | kinase |
| **325** | NM_004759.3 | MAPKAPK2 | mitogen-activated protein kinase-activated protein kinase 2 | Nucleus | kinase |
| **326** | NM_015329.3 | MAU2 | MAU2 chromatid cohesion factor homolog (C. elegans) | Nucleus | other |
| **327** | NR_027037.1 | MBNL1-AS1 | MBNL1 antisense RNA 1 | Other | other |
| **328** | NM_015335.3 | MED13L | mediator complex subunit 13-like | Nucleus | other |
| **329** | NM_017592.1 | MED29 | mediator complex subunit 29 | Nucleus | other |
| **330** | NR_002766.2 | MEG3 | maternally expressed 3 (non-protein coding) | Other | other |
| **331** | NM_024086.3 | METTL16 | methyltransferase like 16 | Other | other |
| **332** | NM_014033.3 | METTL7A | methyltransferase like 7A | Other | other |
| **333** | NR_026914.1 | MGC16275 | uncharacterized protein MGC16275 | Other | other |
| **334** | NM_145764.1 | MGST1 | microsomal glutathione S-transferase 1 | Cytoplasm | enzyme |
| **335** | NM_182744.2 | MINOS1-NBL1/NBL1 | neuroblastoma 1, DAN family BMP antagonist | Nucleus | other |
| **336** | NM_002425.2 | MMP10 | matrix metallopeptidase 10 (stromelysin 2) | Extracellular Space | peptidase |
| **337** | NM_022468.4 | MMP25 | matrix metallopeptidase 25 | Extracellular Space | peptidase |
| **338** | NM_001040097.1 | MOSPD3 | motile sperm domain containing 3 | Other | other |
| **339** | NM_022782.2 | MPHOSPH9 | M-phase phosphoprotein 9 | Cytoplasm | other |
| **340** | NM_002437.4 | MPV17 | MpV17 mitochondrial inner membrane protein | Cytoplasm | other |
| **341** | NM_014046.3 | MRPS18B | mitochondrial ribosomal protein S18B | Cytoplasm | other |
| **342** | NM_006745.3 | MSMO1 | methylsterol monooxygenase 1 | Cytoplasm | enzyme |
| **343** | NM_198080.2 | MSRB3 | methionine sulfoxide reductase B3 | Other | other |
| **344** | NM_005953.3 | MT2A | metallothionein 2A | Cytoplasm | other |
| **345** | NM_003828.2 | MTMR1 | myotubularin related protein 1 | Cytoplasm | phosphatase |
| **346** | NM_001161819.1 | MYO1B | myosin IB | Cytoplasm | other |
| **347** | NM_017433.4 | MYO3A | myosin IIIA | Cytoplasm | kinase |
| **348** | NM_133337.2 | MYOF | myoferlin | Nucleus | other |
| **349** | NM_001113202.1 | NACA | nascent polypeptide-associated complex alpha subunit | Cytoplasm | transcription regulator |
| **350** | NM_181351.3 | NCAM1 | neural cell adhesion molecule 1 | Plasma Membrane | other |
| **351** | NR_003186.1 | NCF1B | neutrophil cytosolic factor 1B pseudogene | Other | other |
| **352** | NM_181782.3 | NCOA7 | nuclear receptor coactivator 7 | Nucleus | transcription regulator |
| **353** | NR_026750.1 | NCR3LG1 | natural killer cell cytotoxicity receptor 3 ligand 1 | Other | other |
| **354** | NM_030571.3 | NDFIP1 | Nedd4 family interacting protein 1 | Cytoplasm | other |
| **355** | NM_201535.1 | NDRG2 | NDRG family member 2 | Cytoplasm | other |
| **356** | NM_021076.3 | NEFH | neurofilament, heavy polypeptide | Cytoplasm | other |
| **357** | NM_033116.4 | NEK9 | NIMA-related kinase 9 | Nucleus | kinase |
| **358** | NM_002500.2 | NEUROD1 | neuronal differentiation 1 | Nucleus | transcription regulator |
| **359** | NM_001134673.3 | NFIA | nuclear factor I/A | Nucleus | transcription regulator |
| **360** | NM_014380.1 | NGFRAP1 | nerve growth factor receptor (TNFRSF16) associated protein 1 | Plasma Membrane | other |
| **361** | NM_020202.4 | NIT2 | nitrilase family, member 2 | Cytoplasm | enzyme |
| **362** | NM_004688.2 | NMI | N-myc (and STAT) interactor | Cytoplasm | transcription regulator |
| **363** | NM_006169.2 | NNMT | nicotinamide N-methyltransferase | Cytoplasm | enzyme |
| **364** | NR_024020.1 | NOL8 | nucleolar protein 8 | Nucleus | other |
| **365** | NM_003995.3 | NPR2 | natriuretic peptide receptor B/guanylate cyclase B (atrionatriuretic peptide receptor B) | Plasma Membrane | G-protein coupled receptor |
| **366** | NM_006186.3 | NR4A2 | nuclear receptor subfamily 4, group A, member 2 | Nucleus | ligand-dependent nuclear receptor |
| **367** | NM_001144772.1 | NSMAF | neutral sphingomyelinase (N-SMase) activation associated factor | Cytoplasm | other |
| **368** | NM_018044.3 | NSUN5 | NOP2/Sun domain family, member 5 | Other | other |
| **369** | NM_006179.4 | NTF4 | neurotrophin 4 | Extracellular Space | growth factor |
| **370** | NM_006181.1 | NTN3 | netrin 3 | Extracellular Space | other |
| **371** | NR_002212.3 | NUDT4P1 | nudix (nucleoside diphosphate linked moiety X)-type motif 4 pseudogene 1 | Other | other |
| **372** | NM_020772.2 | NUFIP2 | nuclear fragile X mental retardation protein interacting protein 2 | Cytoplasm | other |
| **373** | NM_139132.3 | NUP98 | nucleoporin 98kDa | Nucleus | transporter |
| **374** | NM_001081491.1 | NXF1 | nuclear RNA export factor 1 | Nucleus | transporter |
| **375** | NM_033014.2 | OGN | osteoglycin | Extracellular Space | growth factor |
| **376** | NM_181672.2 | OGT | O-linked N-acetylglucosamine (GlcNAc) transferase | Cytoplasm | enzyme |
| **377** | NM_145243.3 | OMA1 | OMA1 zinc metallopeptidase | Cytoplasm | peptidase |
| **378** | NM_001004477.1 | OR10X1 | olfactory receptor, family 10, subfamily X, member 1 | Plasma Membrane | G-protein coupled receptor |
| **379** | NM_012364.1 | OR1Q1 | olfactory receptor, family 1, subfamily Q, member 1 | Plasma Membrane | G-protein coupled receptor |
| **380** | NM_001004714.1 | OR4K13 | olfactory receptor, family 4, subfamily K, member 13 | Plasma Membrane | other |
| **381** | NM_001004059.2 | OR4S2 | olfactory receptor, family 4, subfamily S, member 2 | Plasma Membrane | G-protein coupled receptor |
| **382** | NM_001005162.2 | OR52B6 | olfactory receptor, family 52, subfamily B, member 6 | Plasma Membrane | G-protein coupled receptor |
| **383** | NM_001005281.1 | OR6B1 | olfactory receptor, family 6, subfamily B, member 1 | Plasma Membrane | G-protein coupled receptor |
| **384** | NM_020157.2 | OTOR | otoraplin | Extracellular Space | other |
| **385** | NM_001136157.1 | OTUD5 | OTU domain containing 5 | Cytoplasm | enzyme |
| **386** | NM_000430.3 | PAFAH1B1 | platelet-activating factor acetylhydrolase 1b, regulatory subunit 1 (45kDa) | Cytoplasm | enzyme |
| **387** | NM_004670.3 | PAPSS2 | 3'-phosphoadenosine 5'-phosphosulfate synthase 2 | Cytoplasm | enzyme |
| **388** | NM_015393.2 | PARM1 | prostate androgen-regulated mucin-like protein 1 | Extracellular Space | other |
| **389** | NM_016631.3 | PAXBP1 | PAX3 and PAX7 binding protein 1 | Nucleus | other |
| **390** | NM_181042.3 | PBRM1 | polybromo 1 | Nucleus | other |
| **391** | NM_000282.3 | PCCA | propionyl CoA carboxylase, alpha polypeptide | Cytoplasm | enzyme |
| **392** | NM_031864.1 | PCDHA12 | protocadherin alpha 12 | Other | other |
| **393** | NM_019120.2 | PCDHB8 | protocadherin beta 8 | Plasma Membrane | other |
| **394** | NM_032223.2 | PCNXL3 | pecanex-like 3 (Drosophila) | Other | other |
| **395** | NM_006198.2 | PCP4 | Purkinje cell protein 4 | Cytoplasm | other |
| **396** | NM_016297.3 | PCYOX1 | prenylcysteine oxidase 1 | Cytoplasm | enzyme |
| **397** | NM_014891.5 | PDAP1 | PDGFA associated protein 1 | Cytoplasm | other |
| **398** | NM_002609.3 | PDGFRB | platelet-derived growth factor receptor, beta polypeptide | Plasma Membrane | kinase |
| **399** | NM_176871.2 | PDLIM2 | PDZ and LIM domain 2 (mystique) | Cytoplasm | other |
| **400** | NM_006210.2 | PEG3 | paternally expressed 3 | Nucleus | kinase |
| **401** | NM_021965.3 | PGM5 | phosphoglucomutase 5 | Cytoplasm | enzyme |
| **402** | NM_000926.4 | PGR | progesterone receptor | Nucleus | ligand-dependent nuclear receptor |
| **403** | NM_032758.3 | PHF5A | PHD finger protein 5A | Nucleus | transcription regulator |
| **404** | NM_006214.3 | PHYH | phytanoyl-CoA 2-hydroxylase | Cytoplasm | enzyme |
| **405** | NM_017933.4 | PID1 | phosphotyrosine interaction domain containing 1 | Cytoplasm | other |
| **406** | NM_153747.1 | PIGC | phosphatidylinositol glycan anchor biosynthesis, class C | Cytoplasm | enzyme |
| **407** | NM_002648.3 | PIM1 | pim-1 oncogene | Cytoplasm | kinase |
| **408** | NM_031220.3 | PITPNM3 | PITPNM family member 3 | Cytoplasm | transporter |
| **409** | NM_030821.4 | PLA2G12A | phospholipase A2, group XIIA | Other | enzyme |
| **410** | NM_000301.3 | PLG | plasminogen | Extracellular Space | peptidase |
| **411** | NM_001122.2 | PLIN2 | perilipin 2 | Plasma Membrane | other |
| **412** | NM_015993.2 | PLLP | plasmolipin | Plasma Membrane | transporter |
| **413** | NM_007221.2 | PMF1 | polyamine-modulated factor 1 | Nucleus | transcription regulator |
| **414** | NM_015352.1 | POFUT1 | protein O-fucosyltransferase 1 | Cytoplasm | enzyme |
| **415** | NM_019896.2 | POLE4 | polymerase (DNA-directed), epsilon 4, accessory subunit | Nucleus | enzyme |
| **416** | NM_032483.3 | PPAPDC1B | phosphatidic acid phosphatase type 2 domain containing 1B | Other | phosphatase |
| **417** | NM_152542.3 | PPM1K | protein phosphatase, Mg2+/Mn2+ dependent, 1K | Cytoplasm | phosphatase |
| **418** | NM_014225.5 | PPP2R1A | protein phosphatase 2, regulatory subunit A, alpha | Cytoplasm | phosphatase |
| **419** | NM_021131.3 | PPP2R4 | protein phosphatase 2A activator, regulatory subunit 4 | Cytoplasm | phosphatase |
| **420** | NM_002574.2 | PRDX1 | peroxiredoxin 1 | Cytoplasm | enzyme |
| **421** | NM_014098.2 | PRDX3 | peroxiredoxin 3 | Cytoplasm | enzyme |
| **422** | NM_013388.4 | PREB | prolactin regulatory element binding | Nucleus | transcription regulator |
| **423** | NM_002734.3 | PRKAR1A | protein kinase, cAMP-dependent, regulatory, type I, alpha | Cytoplasm | kinase |
| **424** | NM_002736.2 | PRKAR2B | protein kinase, cAMP-dependent, regulatory, type II, beta | Cytoplasm | kinase |
| **425** | NM_006255.3 | PRKCH | protein kinase C, eta | Cytoplasm | kinase |
| **426** | NM_017892.3 | PRPF40A | PRP40 pre-mRNA processing factor 40 homolog A (S. cerevisiae) | Nucleus | other |
| **427** | NM_007173.4 | PRSS23 | protease, serine, 23 | Extracellular Space | peptidase |
| **428** | NM_015225.2 | PRUNE2 | prune homolog 2 (Drosophila) | Other | other |
| **429** | NM_002778.2 | PSAP | prosaposin | Extracellular Space | other |
| **430** | NM_002791.1 | PSMA6 | proteasome (prosome, macropain) subunit, alpha type, 6 | Cytoplasm | peptidase |
| **431** | NM_000314.4 | PTEN | phosphatase and tensin homolog | Cytoplasm | phosphatase |
| **432** | NM_002834.3 | PTPN11 | protein tyrosine phosphatase, non-receptor type 11 | Cytoplasm | phosphatase |
| **433** | NM_130440.2 | PTPRF | protein tyrosine phosphatase, receptor type, F | Plasma Membrane | phosphatase |
| **434** | NM_005859.3 | PURA | purine-rich element binding protein A | Nucleus | transcription regulator |
| **435** | NR_022011.1 | PWARSN | Prader Willi/Angelman region RNA, SNRPN neighbor | Other | other |
| **436** | NM_198928.3 | PYHIN1 | pyrin and HIN domain family, member 1 | Nucleus | other |
| **437** | NM_000320.2 | QDPR | quinoid dihydropteridine reductase | Cytoplasm | enzyme |
| **438** | NM_004663.3 | RAB11A | RAB11A, member RAS oncogene family | Cytoplasm | enzyme |
| **439** | NM_014904.2 | RAB11FIP2 | RAB11 family interacting protein 2 (class I) | Cytoplasm | other |
| **440** | NM_014700.3 | RAB11FIP3 | RAB11 family interacting protein 3 (class II) | Cytoplasm | other |
| **441** | NM_001142624.2 | RAB34 | RAB34, member RAS oncogene family | Cytoplasm | enzyme |
| **442** | NM_002868.2 | RAB5B | RAB5B, member RAS oncogene family | Cytoplasm | enzyme |
| **443** | NM_004582.2 | RABGGTB | Rab geranylgeranyltransferase, beta subunit | Cytoplasm | enzyme |
| **444** | NM_015646.4 | RAP1B | RAP1B, member of RAS oncogene family | Cytoplasm | enzyme |
| **445** | NM_001098531.2 | RAPGEF3 | Rap guanine nucleotide exchange factor (GEF) 3 | Nucleus | other |
| **446** | NM_031229.2 | RBCK1 | RanBP-type and C3HC4-type zinc finger containing 1 | Cytoplasm | transcription regulator |
| **447** | NM_021239.2 | RBM25 | RNA binding motif protein 25 | Nucleus | other |
| **448** | NM_002897.4 | RBMS1 | RNA binding motif, single stranded interacting protein 1 | Nucleus | other |
| **449** | NM_002899.3 | RBP1 | retinol binding protein 1, cellular | Extracellular Space | transporter |
| **450** | NM_001136225.1 | RCOR3 | REST corepressor 3 | Nucleus | other |
| **451** | NM_052862.3 | RCSD1 | RCSD domain containing 1 | Other | other |
| **452** | NM_016316.2 | REV1 | REV1, polymerase (DNA directed) | Nucleus | enzyme |
| **453** | NM_002920.3 | RFX4 | regulatory factor X, 4 (influences HLA class II expression) | Nucleus | transcription regulator |
| **454** | NM_138328.2 | RHBDL3 | rhomboid, veinlet-like 3 (Drosophila) | Plasma Membrane | peptidase |
| **455** | NM_005059.2 | RLN2 | relaxin 2 | Extracellular Space | other |
| **456** | NM_022780.3 | RMND5A | required for meiotic nuclear division 5 homolog A (S. cerevisiae) | Other | other |
| **457** | NM_001004333.3 | RNASEK | ribonuclease, RNase K | Other | peptidase |
| **458** | NM_017610.6 | RNF111 | ring finger protein 111 | Nucleus | enzyme |
| **459** | NM_018434.4 | RNF130 | ring finger protein 130 | Cytoplasm | peptidase |
| **460** | NM_000975.2 | RPL11 | ribosomal protein L11 | Cytoplasm | other |
| **461** | NM_033251.1 | RPL13 | ribosomal protein L13 | Cytoplasm | other |
| **462** | NM_000988.3 | RPL27 | ribosomal protein L27 | Cytoplasm | other |
| **463** | NM_000998.4 | RPL37A | ribosomal protein L37a | Cytoplasm | other |
| **464** | NM_001000.2 | RPL39 | ribosomal protein L39 | Cytoplasm | other |
| **465** | NM_002950.3 | RPN1 | ribophorin I | Cytoplasm | enzyme |
| **466** | NM_001025.4 | RPS23 | ribosomal protein S23 | Cytoplasm | translation regulator |
| **467** | NM_001030.4 | RPS27 | ribosomal protein S27 | Cytoplasm | other |
| **468** | NM_001135592.2 | RPS27A | ribosomal protein S27a | Cytoplasm | other |
| **469** | NM_001031.4 | RPS28 | ribosomal protein S28 | Cytoplasm | other |
| **470** | NM_004165.2 | RRAD | Ras-related associated with diabetes | Cytoplasm | enzyme |
| **471** | NM_001130709.1 | RUFY3 | RUN and FYVE domain containing 3 | Plasma Membrane | other |
| **472** | NM_152682.2 | RWDD4 | RWD domain containing 4 | Other | other |
| **473** | NM_002970.2 | SAT1 | spermidine/spermine N1-acetyltransferase 1 | Cytoplasm | enzyme |
| **474** | NM_002970.2 | SAT1 | spermidine/spermine N1-acetyltransferase 1 | Cytoplasm | enzyme |
| **475** | NM_006918.4 | SC5D | sterol-C5-desaturase | Cytoplasm | enzyme |
| **476** | NM_005505.4 | SCARB1 | scavenger receptor class B, member 1 | Plasma Membrane | transporter |
| **477** | NM_005063.4 | SCD | stearoyl-CoA desaturase (delta-9-desaturase) | Cytoplasm | enzyme |
| **478** | NM_006998.3 | SCGN | secretagogin, EF-hand calcium binding protein | Cytoplasm | other |
| **479** | NM_001165963.1 | SCN1A | sodium channel, voltage-gated, type I, alpha subunit | Plasma Membrane | ion channel |
| **480** | NM_002998.3 | SDC2 | syndecan 2 | Plasma Membrane | other |
| **481** | NM_014300.2 | SEC11A | SEC11 homolog A (S. cerevisiae) | Cytoplasm | peptidase |
| **482** | NM_004206.2 | SEC22C | SEC22 vesicle trafficking protein homolog C (S. cerevisiae) | Cytoplasm | other |
| **483** | NM_003005.3 | SELP | selectin P (granule membrane protein 140kDa, antigen CD62) | Plasma Membrane | transmembrane receptor |
| **484** | NM_001011553.2 | SEPT7 | septin 7 | Cytoplasm | other |
| **485** | NM_014159.6 | SETD2 | SET domain containing 2 | Cytoplasm | enzyme |
| **486** | NM_001080517.1 | SETD5 | SET domain containing 5 | Other | other |
| **487** | NM_138484.2 | SGOL1 | shugoshin-like 1 (S. pombe) | Nucleus | other |
| **488** | NM_030791.2 | SGPP1 | sphingosine-1-phosphate phosphatase 1 | Cytoplasm | phosphatase |
| **489** | NM_030791.2 | SGPP1 | sphingosine-1-phosphate phosphatase 1 | Cytoplasm | phosphatase |
| **490** | NM_004844.3 | SH3BP5 | SH3-domain binding protein 5 (BTK-associated) | Cytoplasm | other |
| **491** | NM_001017995.2 | SH3PXD2B | SH3 and PX domains 2B | Cytoplasm | other |
| **492** | NM_001098612.1 | SIGLEC14 | sialic acid binding Ig-like lectin 14 | Other | other |
| **493** | NM_014442.2 | SIGLEC8 | sialic acid binding Ig-like lectin 8 | Plasma Membrane | transmembrane receptor |
| **494** | NM_012241.3 | SIRT5 | sirtuin 5 | Cytoplasm | enzyme |
| **495** | NM_213606.3 | SLC16A12 | solute carrier family 16, member 12 (monocarboxylic acid transporter 12) | Other | other |
| **496** | NM_004171.3 | SLC1A2 | solute carrier family 1 (glial high affinity glutamate transporter), member 2 | Plasma Membrane | transporter |
| **497** | NM_003038.3 | SLC1A4 | solute carrier family 1 (glutamate/neutral amino acid transporter), member 4 | Plasma Membrane | transporter |
| **498** | NM_022810.1 | SLC25A14 | solute carrier family 25 (mitochondrial carrier, brain), member 14 | Cytoplasm | transporter |
| **499** | NM_001006641.1 | SLC25A25 | solute carrier family 25 (mitochondrial carrier; phosphate carrier), member 25 | Cytoplasm | transporter |
| **500** | NM_001152.4 | SLC25A5 | solute carrier family 25 (mitochondrial carrier; adenine nucleotide translocator), member 5 | Cytoplasm | transporter |
| **501** | NM_001078175.1 | SLC29A1 | solute carrier family 29 (nucleoside transporters), member 1 | Plasma Membrane | transporter |
| **502** | NM_006516.2 | SLC2A1 | solute carrier family 2 (facilitated glucose transporter), member 1 | Plasma Membrane | transporter |
| **503** | NM_007001.2 | SLC35D2 | solute carrier family 35, member D2 | Cytoplasm | transporter |
| **504** | NM_152462.2 | SLC35G3 | solute carrier family 35, member G3 | Other | enzyme |
| **505** | NM_018976.4 | SLC38A2 | solute carrier family 38, member 2 | Plasma Membrane | transporter |
| **506** | NM_012319.3 | SLC39A6 | solute carrier family 39 (zinc transporter), member 6 | Plasma Membrane | transporter |
| **507** | NM_003048.3 | SLC9A2 | solute carrier family 9, subfamily A (NHE2, cation proton antiporter 2), member 2 | Plasma Membrane | transporter |
| **508** | NM_001184749.1 | SLITRK4 | SLIT and NTRK-like family, member 4 | Extracellular Space | other |
| **509** | NM_001145103.1 | SMAD3 | SMAD family member 3 | Nucleus | transcription regulator |
| **510** | NM_001124767.1 | SMIM4 | small integral membrane protein 4 | Other | other |
| **511** | NR_024399.1 | SNAI3-AS1 | SNAI3 antisense RNA 1 | Other | other |
| **512** | NM_003095.2 | SNRPF | small nuclear ribonucleoprotein polypeptide F | Nucleus | other |
| **513** | NM_013306.3 | SNX15 | sorting nexin 15 | Cytoplasm | transporter |
| **514** | NR_002939.2 | SNX29P2 | sorting nexin 29 pseudogene 2 | Other | other |
| **515** | NM_003877.3 | SOCS2 | suppressor of cytokine signaling 2 | Cytoplasm | other |
| **516** | NM_003955.3 | SOCS3 | suppressor of cytokine signaling 3 | Cytoplasm | phosphatase |
| **517** | NM_000454.4 | SOD1 | superoxide dismutase 1, soluble | Cytoplasm | enzyme |
| **518** | NM_033326.3 | SOX6 | SRY (sex determining region Y)-box 6 | Nucleus | transcription regulator |
| **519** | NM_003111.4 | SP3 | Sp3 transcription factor | Nucleus | transcription regulator |
| **520** | NM_004684.4 | SPARCL1 | SPARC-like 1 (hevin) | Extracellular Space | other |
| **521** | NM_032261.4 | SPATC1L | spermatogenesis and centriole associated 1-like | Other | other |
| **522** | NM_005842.2 | SPRY2 | sprouty homolog 2 (Drosophila) | Plasma Membrane | other |
| **523** | NM_178313.2 | SPTBN1 | spectrin, beta, non-erythrocytic 1 | Plasma Membrane | other |
| **524** | NM_178324.1 | SPTLC1 | serine palmitoyltransferase, long chain base subunit 1 | Cytoplasm | enzyme |
| **525** | NM_152546.2 | SRFBP1 | serum response factor binding protein 1 | Nucleus | other |
| **526** | NM_001130440.1 | SRP9 | signal recognition particle 9kDa | Cytoplasm | other |
| **527** | NM_014467.2 | SRPX2 | sushi-repeat containing protein, X-linked 2 | Cytoplasm | other |
| **528** | NM_006925.3 | SRSF5 | serine/arginine-rich splicing factor 5 | Nucleus | other |
| **529** | NM_003142.3 | SSB | Sjogren syndrome antigen B (autoantigen La) | Nucleus | enzyme |
| **530** | NM_003144.3 | SSR1 | signal sequence receptor, alpha | Cytoplasm | other |
| **531** | NM_152996.2 | ST6GALNAC3 | ST6 (alpha-N-acetyl-neuraminyl-2,3-beta-galactosyl-1,3)-N-acetylgalactosaminide alpha-2,6-sialyltransferase 3 | Cytoplasm | enzyme |
| **532** | NM_003034.3 | ST8SIA1 | ST8 alpha-N-acetyl-neuraminide alpha-2,8-sialyltransferase 1 | Cytoplasm | enzyme |
| **533** | NM_005862.2 | STAG1 | stromal antigen 1 | Nucleus | other |
| **534** | NM_000349.2 | STAR | steroidogenic acute regulatory protein | Cytoplasm | transporter |
| **535** | NM_004226.3 | STK17B | serine/threonine kinase 17b | Nucleus | kinase |
| **536** | NM_015690.3 | STK36 | serine/threonine kinase 36 | Cytoplasm | kinase |
| **537** | NM_177424.2 | STX12 | syntaxin 12 | Cytoplasm | other |
| **538** | NM_024670.3 | SUV39H2 | suppressor of variegation 3-9 homolog 2 (Drosophila) | Nucleus | transcription regulator |
| **539** | NM_033025.4 | SYDE1 | synapse defective 1, Rho GTPase, homolog 1 (C. elegans) | Cytoplasm | other |
| **540** | NM_182910.2 | SYNE2 | spectrin repeat containing, nuclear envelope 2 | Nucleus | other |
| **541** | NM_006772.2 | SYNGAP1 | synaptic Ras GTPase activating protein 1 | Plasma Membrane | other |
| **542** | NM_015093.4 | TAB2 | TGF-beta activated kinase 1/MAP3K7 binding protein 2 | Cytoplasm | other |
| **543** | NM_004606.3 | TAF1 | TAF1 RNA polymerase II, TATA box binding protein (TBP)-associated factor, 250kDa | Nucleus | transcription regulator |
| **544** | NM_006951.3 | TAF5 | TAF5 RNA polymerase II, TATA box binding protein (TBP)-associated factor, 100kDa | Nucleus | transcription regulator |
| **545** | NM_001025247.1 | TAF5L | TAF5-like RNA polymerase II, p300/CBP-associated factor (PCAF)-associated factor, 65kDa | Nucleus | transcription regulator |
| **546** | NM_000593.5 | TAP1 | transporter 1, ATP-binding cassette, sub-family B (MDR/TAP) | Cytoplasm | transporter |
| **547** | NM_018309.2 | TBC1D23 | TBC1 domain family, member 23 | Other | other |
| **548** | NM_152380.2 | TBX15 | T-box 15 | Nucleus | transcription regulator |
| **549** | NM_198795.1 | TDRD1 | tudor domain containing 1 | Cytoplasm | other |
| **550** | NM_138501.4 | TECR | trans-2,3-enoyl-CoA reductase | Plasma Membrane | enzyme |
| **551** | NM_170754.2 | TENC1 | tensin like C1 domain containing phosphatase (tensin 2) | Plasma Membrane | other |
| **552** | NM_016020.2 | TFB1M | transcription factor B1, mitochondrial | Cytoplasm | transcription regulator |
| **553** | NM_006521.4 | TFE3 | transcription factor binding to IGHM enhancer 3 | Nucleus | transcription regulator |
| **554** | NM_005119.3 | THRAP3 | thyroid hormone receptor associated protein 3 | Nucleus | transcription regulator |
| **555** | NM_000461.4 | THRB | thyroid hormone receptor, beta | Nucleus | ligand-dependent nuclear receptor |
| **556** | NM_000362.4 | TIMP3 | TIMP metallopeptidase inhibitor 3 | Extracellular Space | other |
| **557** | NM_003268.5 | TLR5 | toll-like receptor 5 | Plasma Membrane | transmembrane receptor |
| **558** | NM_003273.2 | TM7SF2 | transmembrane 7 superfamily member 2 | Cytoplasm | enzyme |
| **559** | NM_001080506.1 | TMEM150C | transmembrane protein 150C | Other | other |
| **560** | NM_001161342.1 | TMEM171 | transmembrane protein 171 | Other | other |
| **561** | NM_032842.3 | TMEM209 | transmembrane protein 209 | Other | other |
| **562** | NM_001080546.1 | TMEM218 | transmembrane protein 218 | Extracellular Space | other |
| **563** | NM_032012.3 | TMEM245 | transmembrane protein 245 | Other | other |
| **564** | NM_015012.2 | TMEM41B | transmembrane protein 41B | Other | other |
| **565** | NM_006134.5 | TMEM50B | transmembrane protein 50B | Plasma Membrane | other |
| **566** | NM_006290.2 | TNFAIP3 | tumor necrosis factor, alpha-induced protein 3 | Nucleus | enzyme |
| **567** | NM_003842.4 | TNFRSF10B | tumor necrosis factor receptor superfamily, member 10b | Plasma Membrane | transmembrane receptor |
| **568** | NM_002270.3 | TNPO1 | transportin 1 | Nucleus | transporter |
| **569** | NM_014494.2 | TNRC6A | trinucleotide repeat containing 6A | Nucleus | other |
| **570** | NM_003286.2 | TOP1 | topoisomerase (DNA) I | Nucleus | enzyme |
| **571** | NM_005427.2 | TP73 | tumor protein p73 | Nucleus | transcription regulator |
| **572** | NM_025228.2 | TRAF3IP3 | TRAF3 interacting protein 3 | Other | other |
| **573** | NM_016209.3 | TRAPPC2L | trafficking protein particle complex 2-like | Cytoplasm | other |
| **574** | NM_021643.3 | TRIB2 | tribbles homolog 2 (Drosophila) | Plasma Membrane | kinase |
| **575** | NM_001024940.2 | TRIM17 | tripartite motif containing 17 | Cytoplasm | enzyme |
| **576** | NM_152620.2 | TRIM60 | tripartite motif containing 60 | Other | other |
| **577** | NM_007032.5 | TRIOBP | TRIO and F-actin binding protein | Nucleus | other |
| **578** | NM_152307.2 | TRMT61A | tRNA methyltransferase 61 homolog A (S. cerevisiae) | Other | enzyme |
| **579** | NM_016179.2 | TRPC4 | transient receptor potential cation channel, subfamily C, member 4 | Plasma Membrane | ion channel |
| **580** | NM_020389.2 | TRPC7 | transient receptor potential cation channel, subfamily C, member 7 | Plasma Membrane | ion channel |
| **581** | NM_001033678.3 | TRPT1 | tRNA phosphotransferase 1 | Other | enzyme |
| **582** | NM_004089.3 | TSC22D3 | TSC22 domain family, member 3 | Nucleus | transcription regulator |
| **583** | NM_005723.3 | TSPAN5 | tetraspanin 5 | Plasma Membrane | other |
| **584** | NM_014639.2 | TTC37 | tetratricopeptide repeat domain 37 | Other | other |
| **585** | NM_001029964.2 | TTLL13 | tubulin tyrosine ligase-like family, member 13 | Other | enzyme |
| **586** | NM_024331.3 | TTPAL | tocopherol (alpha) transfer protein-like | Other | other |
| **587** | NM_006009.2 | TUBA1A | tubulin, alpha 1a | Cytoplasm | other |
| **588** | NM_015914.5 | TXNDC11 | thioredoxin domain containing 11 | Cytoplasm | enzyme |
| **589** | NM_032731.3 | TXNDC17 | thioredoxin domain containing 17 | Cytoplasm | enzyme |
| **590** | NM_198329.2 | UBA5 | ubiquitin-like modifier activating enzyme 5 | Cytoplasm | enzyme |
| **591** | NM_003344.2 | UBE2H | ubiquitin-conjugating enzyme E2H | Other | enzyme |
| **592** | NM_001145161.1 | UBE2QL1 | ubiquitin-conjugating enzyme E2Q family-like 1 | Other | other |
| **593** | NM_006798.2 | UGT2A1 | UDP glucuronosyltransferase 2 family, polypeptide A1, complex locus | Cytoplasm | enzyme |
| **594** | NM_006830.3 | UQCR11 | ubiquinol-cytochrome c reductase, complex III subunit XI | Cytoplasm | enzyme |
| **595** | NM_001089591.1 | UQCRHL | ubiquinol-cytochrome c reductase hinge protein-like | Other | other |
| **596** | NM_018218.2 | USP40 | ubiquitin specific peptidase 40 | Other | peptidase |
| **597** | NM_007124.2 | UTRN | utrophin | Plasma Membrane | transmembrane receptor |
| **598** | NM_018445.4 | VIMP | VCP-interacting membrane protein | Cytoplasm | other |
| **599** | NM_030938.3 | VMP1 | vacuole membrane protein 1 | Plasma Membrane | other |
| **600** | NM_182546.2 | VSTM2A | V-set and transmembrane domain containing 2A | Extracellular Space | other |
| **601** | NM_006646.5 | WASF3 | WAS protein family, member 3 | Cytoplasm | other |
| **602** | NM_001006657.1 | WDR35 | WD repeat domain 35 | Cytoplasm | other |
| **603** | NM_001006657.1 | WDR35 | WD repeat domain 35 | Cytoplasm | other |
| **604** | NM_139281.2 | WDR36 | WD repeat domain 36 | Extracellular Space | other |
| **605** | NM_015285.2 | WDR7 | WD repeat domain 7 | Other | other |
| **606** | NM_001080435.1 | WHAMM | WAS protein homolog associated with actin, golgi membranes and microtubules | Cytoplasm | other |
| **607** | NM_003882.2 | WISP1 | WNT1 inducible signaling pathway protein 1 | Extracellular Space | other |
| **608** | NM_003881.2 | WISP2 | WNT1 inducible signaling pathway protein 2 | Extracellular Space | growth factor |
| **609** | NM_003392.3 | WNT5A | wingless-type MMTV integration site family, member 5A | Extracellular Space | cytokine |
| **610** | NM_152857.1 | WTAP | Wilms tumor 1 associated protein | Nucleus | other |
| **611** | NM_015691.3 | WWC3 | WWC family member 3 | Other | other |
| **612** | NM_007013.3 | WWP1 | WW domain containing E3 ubiquitin protein ligase 1 | Cytoplasm | enzyme |
| **613** | NM_014950.2 | ZBTB1 | zinc finger and BTB domain containing 1 | Nucleus | other |
| **614** | NM_005453.4 | ZBTB22 | zinc finger and BTB domain containing 22 | Nucleus | other |
| **615** | NM_025079.2 | ZC3H12A | zinc finger CCCH-type containing 12A | Cytoplasm | other |
| **616** | NM_033089.6 | ZCCHC3 | zinc finger, CCHC domain containing 3 | Other | other |
| **617** | NM_016107.3 | ZFR | zinc finger RNA binding protein | Nucleus | other |
| **618** | NM_004799.2 | ZFYVE9 | zinc finger, FYVE domain containing 9 | Cytoplasm | peptidase |
| **619** | NM_001012981.4 | ZKSCAN2 | zinc finger with KRAB and SCAN domains 2 | Nucleus | transcription regulator |
| **620** | NM_020338.3 | ZMIZ1 | zinc finger, MIZ-type containing 1 | Nucleus | other |
| **621** | NM_006959.2 | ZNF17 | zinc finger protein 17 | Nucleus | other |
| **622** | NM_001005368.1 | ZNF32 | zinc finger protein 32 | Nucleus | other |
| **623** | NM_018660.2 | ZNF395 | zinc finger protein 395 | Cytoplasm | other |
| **624** | NM_153257.2 | ZNF461 | zinc finger protein 461 | Nucleus | other |
| **625** | NM_001159860.1 | ZNF583 | zinc finger protein 583 | Nucleus | other |
| **626** | NM_152437.1 | ZNF664 | zinc finger protein 664 | Nucleus | other |
| **627** | NM_024836.1 | ZNF672 | zinc finger protein 672 | Other | other |
| **628** | NR_003936.1 | ZNF83 | zinc finger protein 83 | Nucleus | transcription regulator |
| **629** | NM_032268.4 | ZNRF1 | zinc and ring finger 1, E3 ubiquitin protein ligase | Cytoplasm | enzyme |
| **630** | NM_005455.3 | ZRANB2 | zinc finger, RAN-binding domain containing 2 | Nucleus | transcription regulator |
| **631** | NM_032143.2 | ZRANB3 | zinc finger, RAN-binding domain containing 3 | Nucleus | enzyme |
| **632** | NR_024063.1 | ZSCAN12P1 | zinc finger and SCAN domain containing 12 pseudogene 1 | Other | other |
| **633** | NM_152677.2 | ZSCAN4 | zinc finger and SCAN domain containing 4 | Nucleus | other |
| **634** | NR_002801.1 | unknown | unknown | unknown | unknown |
| **635** | XM_002342472.1 | unknown | unknown | unknown | unknown |
| **636** | XM_002347727.1 | unknown | unknown | unknown | unknown |
| **637** | XM_002342539.1 | unknown | unknown | unknown | unknown |
| **638** | XM_002344087.1 | unknown | unknown | unknown | unknown |
| **639** | XM_002347802.1 | unknown | unknown | unknown | unknown |
| **640** | XR_078533.1 | unknown | unknown | unknown | unknown |
| **641** | XM_001725248.1 | unknown | unknown | unknown | unknown |
| **642** | XR_078481.1 | unknown | unknown | unknown | unknown |
| **643** | XR_078968.1 | unknown | unknown | unknown | unknown |
| **644** | XR_037889.2 | unknown | unknown | unknown | unknown |
| **645** | XR_041540.1 | unknown | unknown | unknown | unknown |
| **646** | XM_002343060.1 | unknown | unknown | unknown | unknown |
| **647** | XM_002342023.1 | unknown | unknown | unknown | unknown |
| **648** | XM_001125753.2 | unknown | unknown | unknown | unknown |
| **649** | XR_040546.1 | unknown | unknown | unknown | unknown |
| **650** | XM_002344196. | unknown | unknown | unknown | unknown |
| **651** | XR_078670.1 | unknown | unknown | unknown | unknown |
| **652** | XR_040843.2 | unknown | unknown | unknown | unknown |
| **653** | XM_002344690.1 | unknown | unknown | unknown | unknown |
| **654** | XM_001128221.2 | unknown | unknown | unknown | unknown |
| **655** | XM_002343025.1 | unknown | unknown | unknown | unknown |
| **656** | XM_002345211.1 | unknown | unknown | unknown | unknown |
