# Supplementary material for: Deregulation of sertoli and leydig cells function in patients with klinefelter syndrome as evidenced by testis transcriptome analysis
Source: BMC Genomics. 2015 Mar 7;16(1):156. doi: 10.1186/s12864-015-1356-0 (PMC4362638; doi:10.1186/s12864-015-1356-0)
Supplement: Additional file 4: Table S3. — IPA functional analysis of cluster A transcripts. [file 12864_2015_1356_MOESM4_ESM.docx]

TABLE S3: IPA functional analysis of cluster A transcripts

| **FUNCTIONS** | **P-VALUE** | **MOLECULES** | **NUMBER OF TRANSCRIPTS** |
| --- | --- | --- | --- |
| **DNA Replication, Recombination, and Repair** | 6,61E-06-  4,34E-02 | SMC3,TRIP13,NUSAP1,MIS18A,TOPBP1,PHF13,  RACGAP1,RAN,TNP1,NR2C2,XRCC6BP1,CBX5,  UBE2T,PICK1,FANCD2,KAT6A,CDCA5,BTRC,AGO1,CHMP1A,SGOL1,BRCA1,TDRD1,CLU | 24 |
| **Cell**  **Morphology** | 1,21E-04-  4,83E-02 | PPP1CC,PCNT,PVRL3,PDPK1,VCAN,SPESP1,  FANCD2,TMEM38B,EXOC5,BRCA1,HGS,TDRD1,  LAMA5,NME5,RACGAP1,TNP1,VIP,PHLPP2,PICK1,SLC26A4,DNAI2,ADCY10,CX3CL1,  CBFA2T2,ACVR2A | 25 |
| **Organ Morphology** | 1,21E-04-  4,83E-02 | LAMA5,PPP1CC,USP14,NME5,PVRL3,TNP1,PDPK1,VIP,SOD3,NFATC1,SPESP1,PTGER1,PICK1,FANCD2,SLC26A4,FGFR4,IGFBP3,BRCA1,TDRD1,ACVR2A | 20 |
| **Reproductive System Development and Function** | 1,21E-04-  4,84E-02 | TRIP13,PPP1CC,EIF3H,NME5,TNP1,NR2C2,VIP,  SPESP1,PICK1,FANCD2,SPA17,PRKACG,BTRC,  CSNK2B,GABARAP,BRCA1,TDRD1,CLU,ACVR2A | 19 |
| **Molecular Transport** | 2,69E-04-  4,34E-02 | CUBN,CHRNB1,RAN,GGN,PDPK1,GRIA4,VCAN,  NFATC1,PTGER1,VDAC2,PLCD1,USO1,ZG16,  GOSR1,CHMP1A,HGS,ABCB8,CARTPT,RACGAP1,STX5,STAP1,SLC39A3,VIP,PICK1,SERINC3,  SLC26A4,IGFBP3,SLC7A7,GABARAP,  SCAMP3,CX3CL1,CLU,COLQ,ACTR1A | 34 |
| **Cellular Function and Maintenance** | 3,6E-04-  4,52E-02 | LAMA5,PPP1CC,PCNT,CUBN,STX5,CHRNB1,TNP1,VIP,CBX5,GRIA4,NFATC1,USO1,IL33,SLC26A4,  CSNK2B,DNAI2,BRCA1,SGOL1,CTDNEP1,HGS | 20 |
| **Cell Cycle** | 6,46E-04- 4,83E-02 | PPP1CC,COPS2,MIS18A,HAUS3,RAN,EIF6, NFATC1,FANCD2,CDCA5,CSNK2B,BRCA1,CHMP1, SMC3,TRIP13,NUSAP1,TOPBP1,PHF13,RACGAP1,TNP1,VIP,ARL3,CUL2,KAT6A,IGFBP3,  BTRC,SGOL1,CLU | 27 |

P-values indicate a measure of the likelihood that the association between a set of genes in the dataset and a related function is due to random association. P-values < 0.05 indicate a statistically significant, non-random association. Each high level category shown can contain several lower level categories, then p-values for the higher level category are indicated as a range.
