# Supplementary material for: Deregulation of sertoli and leydig cells function in patients with klinefelter syndrome as evidenced by testis transcriptome analysis
Source: BMC Genomics. 2015 Mar 7;16(1):156. doi: 10.1186/s12864-015-1356-0 (PMC4362638; doi:10.1186/s12864-015-1356-0)
Supplement: Additional file 5: Table S4. — IPA functional analysis of cluster B transcripts. [file 12864_2015_1356_MOESM5_ESM.docx]

TABLE S4: IPA functional analysis of cluster B transcripts

| **FUNCTIONS** | **P-VALUE** | **MOLECULES** | **NUMBER OF**  **TRANSCRIPTS** |
| --- | --- | --- | --- |
| **Cell Death and Survival** | 1,23E-07-9,76E-03 | ZC3H12A,MAGED1,KRT6A,KDM6B,DPYD,COX8A,  TAB2,APIP,GMFG,IDE,DYRK3,CCNG1,BNIP3,LCAT,  CAV1,TDRD1,ITGB5,ICOSLG,PDGFRB,ETS1,  ARHGAP10,SLC2A1,RPL27,DCN,TNFRSF10B,NEFH,  DIO3,ASAH1,CTSL1,PPP2R4,APH1A,BTG2,RAB11A,  THRB,SRSF5,GSTP1,SPTBN1,RAP1B,MAP2K6,PRDX, MED29,GFRA3,SOCS2,MTMR1,RLN2,CTNNA1,CEBP,RNF130,AKAP7,TAP1,OGT,SYNGAP1,JUN,GLP2R, ANXA5,MAL,IFI6,EEF2K,PURA,CITED2,NEUROD1, CLN3,ITGA2,MMP10,C5,SLC29A1,SH3BP5,FUS,CSTB,INPP5D,LTBP1,STAR,LDLR,TUBA1A,NR4A2,SOX6, LY9,REV1,CIRBP,LIMS1,SPRY2,ICOS,PSAP,GABPA, BNIP3L,PAFAH1B1,TFB1M,NCAM1,MT2A,SOCS3,JMY,GDA,NTF4,NDRG2,TP73,SMAD3,MAP1B,GIMAP5, LIMK2,CXCR1,DNAJC15,RBP1,TSC22D3,PTEN,PGR, CTBP1,TOP1,WTAP,SCARB1,PIM1,VIMP,LYNX1, ITPRIP,TRIB2,NSMAF,KLF2,STK17B,PEG3,MAP3K14, IGFBP6,PSMA6,MGST1,RRAD,DIDO1,RAPGEF3,GSN,FOXC1,INHBA,APC,PRDX3,PPP2R1A,ADAM12, PTPN11,SELP,ZFR,DUSP1,BBS2,NGFRAP1,PRKCH, SGPP1,B2M,SCD,RBM25,DNASE1L3,CHSY1,SAT1, TNFAIP3,ERC1,BCAR1,SLC25A5,PTPRF,HIC1, LAMTOR3,SP3,TNRC6A,SDC2,ANXA1,RABGGTB, MAPKAPK2,HNRNPC,SIGLEC8,TIMP3,GSTA2, ST8SIA1,SOD1,LUC7L3,NCR3LG1,CD6,CEBPB,PLG, RBCK1,DNM1,TENC1,PRKAR2B,HYPK,SLC1A2, WISP1,CYB5A,EIF2AK3,WNT5A,PRKAR1A | 181 |
| **Lipid Metabolism** | 2,39E-07-9,97E-03 | FTL,IDE,PPAPDC1B,BNIP3,LCAT,CAV1,LSS,OMA1, HSD17B1,PDGFRB,GRIK1,HSD17B3,SLC2A1,MSMO1,ASAH1,HSD17B6,LYPLA2,DLD,THRB,GSTP1,KDSR, GALC,MTMR1,CEBPD,TECR,H2AFZ,LIPA,JUN,PLIN2, ANXA5,CAV2,CITED2,ABHD5,CLN3,C5,TRPC4, SH3BP5,INPP5D,STAR,GLTP,LDLR,NR4A2,GABPA, PSAP,PIGC,AOX1,PAFAH1B1,NCAM1,SOCS3, AKR1C3,SMAD3,CXCR1,RBP1,TSC22D3,SC5D,PTEN,SCARB1,NSMAF,MAP3K14,ACSL3,CYB5B,RRAD, ST6GALNAC3,GSN,SLC38A2,INHBA,APC,PRDX3, PTPN11,DUSP1,SGPP1,SCD,APOH,MAMLD1,SAT1, ANXA1,PHYH,CYGB,MAPKAPK2,HSD17B4,PITPNM3,GSTA2,SPTLC1,ABCD3,ST8SIA1,SOD1,FDX1,CEBPB,LPGAT1,PLG,PRKAR2B,PCCA,TLR5,CLDN16,RBMS1,SLC1A2,EIF2AK3 | 97 |
| **Small Molecule Biochemistry** | 2,39E-07-9,97E-03 | FTL,COX8A,IDE,PPAPDC1B,BNIP3,LCAT,CAV1,LSS, OMA1,HSD17B1,GRIK1,PDGFRB,HSD17B3,SLC2A1, MSMO1,DIO3,ASAH1,HSD17B6,LYPLA2,PPP2R4, NDFIP1,DLD,THRB,NPR2,GSTP1,KDSR,GALC,CEBPD,MTMR1,TECR,H2AFZ,TAP1,LIPA,JUN,PLIN2,ANXA5, CAV2,CITED2,CLN3,ABHD5,IMPDH2,SLC39A6,TRPC4,C5,SH3BP5,INPP5D,STAR,GLTP,NR4A2,LDLR,PSAP,GABPA,PIGC,AOX1,PAFAH1B1,NCAM1,MT2A,SOCS3,AKR1C3,ARL6IP5,SMAD3,CXCR1,RBP1,TSC22D3, SC5D,PTEN,PGR,SCARB1,NSMAF,MAP3K14,ACSL3, CYB5B,RRAD,AK3,ST6GALNAC3,GSN,SLC38A2,APC, INHBA,ATP5C1,PRDX3,PTPN11,DUSP1,B3GAT1, SGPP1,ATP6V0C,SCD,SLC1A4,APOH,MAMLD1,SAT1,SLC25A5,ANXA1,PHYH,CYGB,MAPKAPK2,HSD17B4, PITPNM3,GSTA2,SPTLC1,ABCD3,ST8SIA1,SOD1, FDX1,PID1,CLPX,GLCE,CEBPB,LPGAT1,PLG, PRKAR2B,PCCA,TLR5,CLDN16,SLC1A2, RBMS1,EIF2AK3 | 117 |
| **Cellular Development** | 6,71E-06-9,59E-03 | ZC3H12A,FTL,MAGED1,TAB2,WWP1,IDE,DYRK3, CCNG1,BNIP3,SRPX2,LCAT,CAV1,ITGB5,ICOSLG, PDGFRB,ETS1,SLC2A1,DCN,TNFRSF10B,NEFH, ASAH1,NDFIP1,BTG2,RAB11A,NUP98,THRB,SRSF5, GSTP1,MAP2K6,SPTBN1,RAP1B,MAP2,SOCS2, HNRNPA2B1,CEBPD,RLN2,CTHRC1,PYHIN1,H2AFZ, SYNGAP1,OGT,KIAA1598,JUN,MAL,MEG3,EEF2K, PURA,CITED2,NEUROD1,ABHD5,CLN3,ITGA2,MMP10,C5,FUS,INPP5D,GIT2,ZNF32,LTBP1,TUBA1A,LDLR, NR4A2,MAGI1,SOX6,LIMS1,CIRBP,LAMB3,NFIA, SPRY2,ICOS,VMP1,GABPA,PAFAH1B1,RUFY3,NTN3,NCAM1,MT2A,SOCS3,GDA,NTF4,AKR1C3,TP73, NDRG2,SMAD3,MAP1B,TBX15,LIMK2,CXCR1,RBP1, CD63,TSC22D3,WISP2,PTEN,PGR,CTBP1,TOP1, WTAP,SCARB1,PIM1,TRIB2,TFE3,KLF2,IGFBP6, MAP3K14,EID1,RRAD,SLITRK4,GSN,FOXC1,APC, INHBA,PRDX3,SPARCL1,PPP2R1A,PTPN11,SELP, COL6A3,DUSP1,B3GAT1,PRKCH,B2M,SCD,SAT1, AUP1,TNFAIP3,BCAR1,HIC1,ZMIZ1,PTPRF,SP3, SH3PXD2B,SDC2,ANXA1,MAPKAPK2,NACA,TIMP3, SOD1,ST8SIA1,C1orf151-NBL1/NBL1, FDX1,CEBPB,RBCK1,PLG,TENC1,PRKAR2B,TLR5, RNF111,SLC1A2,WISP1,HOXD9,EIF2AK3,MAP4K4, PRKAR1A,WNT5A | 154 |
| **Cellular Growth and Proliferation** | 6,71E-06-8,73E-03 | FTL,MAGED1,TAB2,COL8A1,IDE,CCNG1,BNIP3,LCAT,GPRC5C,CAV1,NOL8,ICOSLG,PDGFRB,ETS1, SLC2A1,DCN,TNFRSF10B,CSNK1D,LAMC3,PCYOX1, SSR1,ASAH1,CTSL1,NDFIP1,BTG2,NUP98,THRB, SRSF5,GSTP1,MAP2K6,RAP1B,SPTBN1,GFRA3, PRDX1,HNRNPA2B1,SOCS2,CEBPD,RLN2,CTNNA1, CTHRC1,PYHIN1,H2AFZ,TAP1,CPSF4,OGT,LIPA,JUN,MMP25,GLP2R,PLIN2,GAS8,MEG3,EEF2K,FXYD2, CAV2,PURA,CITED2,NEUROD1,CLN3,IMPDH2,ITGA2,MMP10,TRPC4,HNRNPD,C5,SLC29A1,FUS,INPP5D, LTBP1,LDLR,NR4A2,MAGI1,LY9,SOX6,LIMS1,CIRBP, SPRY2,ICOS,VMP1,PSAP,PAFAH1B1,NCAM1,NTN3, MT2A,SOCS3,NTF4,AKR1C3,ARL6IP5,TP73,NDRG2, SMAD3,CXCR1,ECD,CD63,RBP1,WISP2,TSC22D3, PGR,PTEN,CTBP1,NNMT,TOP1,WTAP,SCARB1,PIM1,TRIB2,TFE3,KLF2,STK17B,MAP3K14,IGFBP6,TSPAN5,RRAD,RAPGEF3,HHIP,MYOF,GSN,FOXC1,APC, INHBA,PPP2R1A,SPARCL1,PRDX3,SCGN,COL6A3, PTPN11,ADAM12,SELP,DUSP1,B3GAT1,NGFRAP1, PRKCH,EBI3,B2M,APOH,SAT1,TNFAIP3,BCAR1, SLC25A5,HIC1,PTPRF,ZMIZ1,SDC2,ANXA1,CYGB, MAPKAPK2,HNRNPC,NACA,TIMP3,GSTA2,PREB, SOD1,ST8SIA1,CD6,FDX1,CEBPB,GLCE,RBCK1,PLG,PDAP1,TENC1,PRKAR2B,TLR5,RNF111,SLC1A2, WISP1,MAP4K4,EIF2AK3,PRKAR1A,WNT5A | 170 |
| **Cell Morphology** | 6,86E-06-9,65E-03 | ZC3H12A,KRT6A,TAB2,BNIP3,CAV1,DYNC2H1, TDRD1,PDGFRB,GRIK1,PLA2G12A,ETS1,STK36, ARHGAP10,DCN,TNFRSF10B,FERMT2,CSNK1D, NEFH,CSPG5,BTG2,DLD,RAB11A,THRB,SPTBN1, RAP1B,MAP2K6,MAP2,PRDX1,WASF3,SOCS2, CEBPD,CTHRC1,TAP1,COL5A1,SYNGAP1,LIPA, KIAA1598,JUN,IFI6,CAV2,PURA,CITED2,NEUROD1, CLN3,ABHD5,IMPDH2,C5,INPP5D,CSTB,LTBP1,STAR,TUBA1A,LDLR,LY9,CIRBP,LIMS1,SPRY2,NFIA,ICOS, PSAP,GABPA,BNIP3L,PAFAH1B1,RUFY3,NTN3, NCAM1,MT2A,GDA,NTF4,TP73,SMAD3,MAP1B, GIMAP5,LIMK2,CXCR1,WISP2,CHST15,SC5D, TSC22D3,PTEN,PGR,TOP1,SCARB1,PIM1,TRIB2, KLF2,ZFYVE9,SYNE2,SLITRK4,DIDO1,ACAP1, RAPGEF3,GSN,APC,INHBA,OGN,PRDX3,SPARCL1, PTPN11,ADAM12,SELP,SLC25A14,DUSP1,BBS2, B3GAT1,PRKCH,EBI3,B2M,ATP6V0C,CHSY1, SLC25A5,BCAR1,PTPRF,SDC2,PHYH,ANXA1,UTRN, MAPKAPK2,HSD17B4,SLC9A2,SCN1A,TIMP3,ABCD3,ST8SIA1,SOD1,C1orf151-NBL1/NBL1, CEBPB, DNM1, PLG,PRKAR2B,KIF5B,EIF2AK3,PRKAR1A,WNT5A | 134 |
| **DevelopmentalDisorder** | 1,86E-05-6E-03 | SMAD3,MAP1B,TBX15,TSC22D3,PTEN,LCAT,SRPX2, PIM1,CAV1,DYNC2H1,KLF2,FOXH1,RRAD,RAPGEF3, LAMC3,FOXC1,INHBA,ASAH1,PAPSS2,PRDX3, PTPN11,DUSP1,BTG2,NPR2,MAP2K6,GALC,CRYGD, CHSY1,TNFAIP3,H2AFZ,PTPRF,COL5A1,LIPA, SH3PXD2B,JUN,SP3,PHYH,SLC16A12,PURA, HSD17B4,CITED2,CLN3,ABHD5,WDR35,SOD1,RFX4, GLCE,CSTB,STAR,TUBA1A,LIMS1,NFIA,SPRY2,PSAP,PAFAH1B1,EIF2AK3,PRKAR1A,WNT5A | 58 |
| **Reproductive System Disease** | 1,68E-04-8,73E-03 | COG4,IDE,PCDHB8,NCOA7,LCAT,CAV1,HSD17B1, ITGB5,PDGFRB,HSD17B3,SLC2A1,DCN,HOOK2, LAMC3,SSR1,HSD17B6,CLCN5,LYPLA2,HIBCH,BTG2,PGM5,SRSF5,GSTP1,SPTBN1,RAP1B,PRDX1, CTNNA1,TRPT1,TECR,ZBTB22,C1R,CEACAM7,JUN, PURA,CAV2,CITED2,NEUROD1,CLN3,IMPDH2,SIRT5, ITGA2,SLC39A6,STAR,TUBA1A,LDLR,HNRNPH1, CIRBP,SPRY2,ICOS,DDX1,LYSMD1,SSB,NCAM1, SOCS3,TP73,NDRG2,IFI35,DNAJC15,CXCR1,RPS23, TSC22D3,WISP2,PGR,PTEN,CTBP1,TOP1,PIM1,TFE3,KLF2,PEG3,MAP3K14,MGST1,POLE4,TRIOBP, SLITRK4,FOXC1,DYNLRB1,PAPSS2,INHBA,APC, PPP2R1A,TAF1,PTPN11,DUSP1,PRKCH,EBI3,HMCN2,B2M,SLC1A4,MAMLD1,SAT1,DDX60L,ZCCHC3, PTPRF,HIC1,TNRC6A,SDC2,ARGLU1,EIF2S3, HSD17B4,CPNE3,DMXL1,GSTA2,TIMP3,NCR3LG1, TMEM245,CEBPB,PLG,KIAA1199,NEK9,WNT5A | 111 |
| **Free Radical Scavenging** | 1,97E-04-8,73E-03 | SOCS3,FTL,ZC3H12A,TP73,PRDX1,SMAD3,SAT1, CXCR1,PTEN,BNIP3,SH3PXD2B,SCARB1,LCAT,PIM1,ANXA1,CAV1,CYGB,PDGFRB,ETS1,SOD1,SLC2A1, DCN,C5,RAPGEF3,GSN,SH3BP5,LTBP1,PRDX3, PRSS23,SELP,DUSP1,SPRY2,BNIP3L | 33 |
| **Molecular Transport** | 2,19E-04-9,76E-03 | KLC1,UQCR11,LCAT,CAV1,OMA1,PDGFRB,GRIK1, SLC2A1,HOOK2,CSNK1D,PCYOX1,PLLP,DIO3,ASAH1,CLCN5,LYPLA2,NDFIP1,RAB11A,THRB,NUP98,NPR2,MAP2K6,SPTBN1,GALC,HNRNPA2B1,CEBPD,H2AFZ,AKAP7,TAP1,LIPA,JUN,ANXA5,PLIN2,MAL,FXYD2, CITED2,NEUROD1,CLN3,ABHD5,HNRNPD,C5,TRPC4,NXF1,SLC29A1,FUS,INPP5D,STAR,GLTP,LDLR,ICOS,PSAP,SOCS3,ARL6IP5,NDRG2,SMAD3,MAP1B,RBP1,TSC22D3,SC5D,PGR,PTEN,SCARB1,VIMP,NSMAF, ACSL3,RRAD,TNPO1,RAPGEF3,GSN,SLC38A2,APC, INHBA,PRDX3,PTPN11,DUSP1,SLC25A14,SGPP1, B2M,SCD,ATP6V0C,SLC1A4,APOH,MAMLD1,SAT1, SLC25A5,CEP290,PHYH,ANXA1,MAPKAPK2, HSD17B4,SLC9A2,RAB11FIP3,CPNE3,SCN1A,ABCD3,SPTLC1,ST8SIA1,SOD1,PID1,CEBPB,LPGAT1,TRPC7,BEST1,PLG,PRKAR2B,TLR5,KIF5B,CLDN16,SLC1A2, RBMS1,EIF2AK3,DUSP16 | 112 |

P-values indicate a measure of the likelihood that the association between a set of genes in the dataset and a related function is due to random association. P-values < 0.05 indicate a statistically significant, non-random association. Each high level category shown can contain several lower level categories, then p-values for the higher level category are indicated as a range.
