# Supplementary figures and images for: Deregulation of sertoli and leydig cells function in patients with klinefelter syndrome as evidenced by testis transcriptome analysis
Source: BMC Genomics. 2015 Mar 7;16(1):156. doi: 10.1186/s12864-015-1356-0 (PMC4362638; doi:10.1186/s12864-015-1356-0)

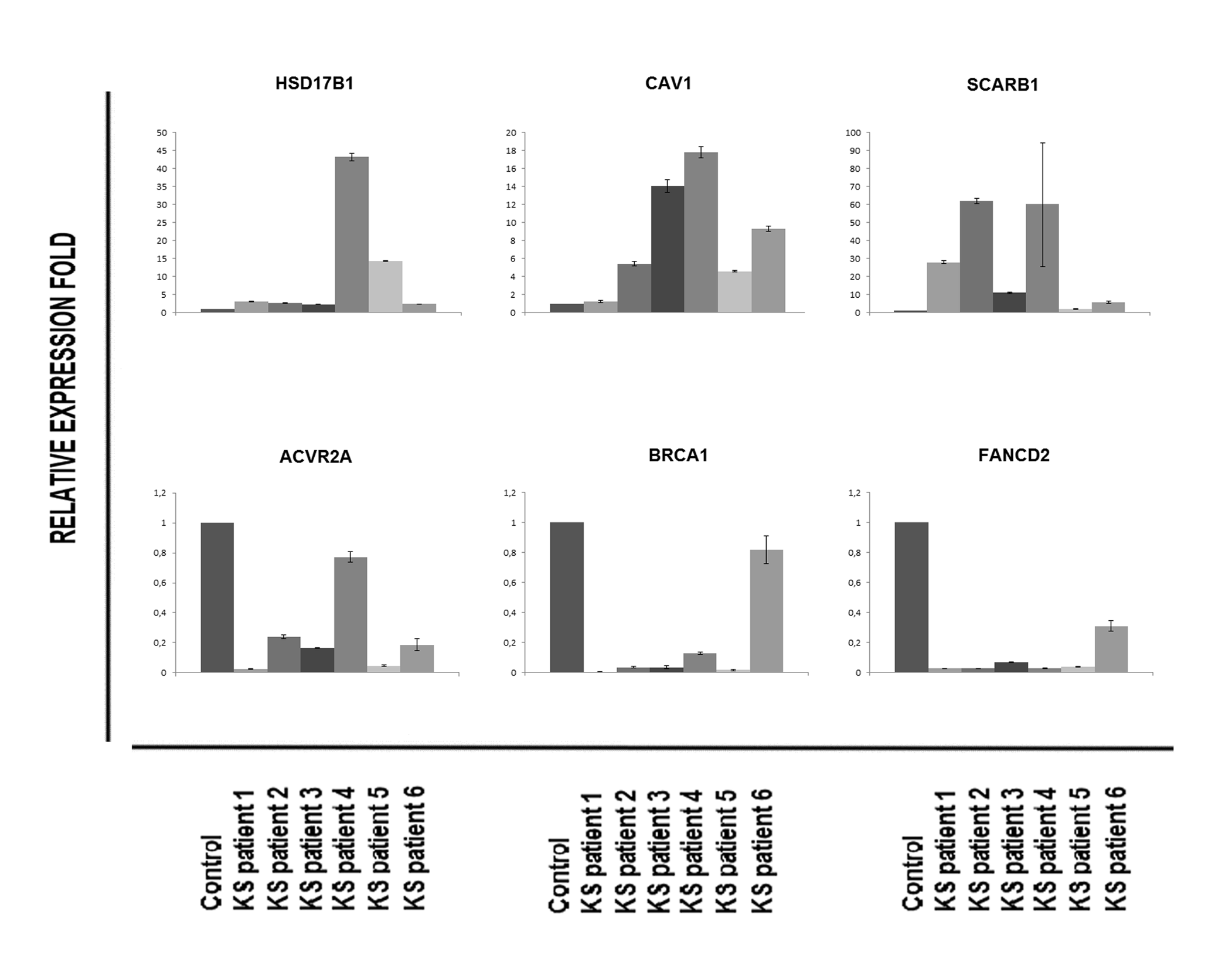

Supplement: Additional file 6: Figure S1. — Validation of microarray gene expression data by qRT-PCR. Bar graph shows mRNA levels of HSD17B1, CAV1, SCARB1, ACVR2A, BRCA1, FANCD2 as measured by quantitative real time PCR in each of KS testis analysed vs. Control testis. qRT-PCR data are expressed as mean values of relative fold changes ± SD of three independent experiments performed in triplicate. qRT-PCR data, for each gene, were significant (p<0.05, Student’s T-test) when compared to Control testis. qRT-PCR data did not show significant difference when compared to MicroArray data (p>0.05, Student’s T-test). [file 12864_2015_1356_MOESM6_ESM.tiff]
